# Generation of thymus-reconstituting T cell progenitors from human pluripotent stem cells

**DOI:** 10.1016/j.crmeth.2025.101272

**Published:** 2026-01-08

**Authors:** Elena S. Philonenko, Baoyun Zhang, Eugene Albert, Zahir Shah, Denis Maksimov, Yahai Shu, Peng Li, Pavel Volchkov, Igor M. Samokhvalov

**Affiliations:** 1 CAS Key Laboratory of Regenerative Biology, Guangdong Provincial Key Laboratory of Stem Cells and Regenerative Medicine, Guangzhou Institutes of Biomedicine and Health, Chinese Academy of Sciences, Guangzhou, China; 2 Federal Research Center for Innovator and Emerging Biomedical and Pharmaceutical Technologies, 125315 Moscow, Russia; 3 Department of Hematology & Hematopoietic Cell Transplantation, City of Hope National Medical Center, Los Angeles, CA 91010, USA; 4 Hematologic Malignancies Research Institute, City of Hope National Medical Center, Los Angeles, CA 91010, USA; 5 Department of Fundamental Medicine, Lomonosov Moscow State University, 119992 Moscow, Russia

**Keywords:** cell transplantation, differentiation, human pluripotent stem cells, T cell progenitors, thymus

## Abstract

Generating a large number of progenitors that can repopulate the immune system of a recipient is one of the key steps toward efficient cancer immunotherapy. Here, we describe the engineering of T cell progenitors capable of direct and long-term reconstitution of the thymus. In the thymus, human pluripotent stem cell (hPSC)-derived progenitor T cells (pro-T cells) developed into single-positive human T cells that entered circulation and settled in the spleen. Single-cell transcriptome analysis of differentiating hPSCs attested to the emergence of cells that displayed the transcription signature of the early T cell progenitors. Comparative transcription profiling revealed the similarity of the hPSC-pro-T cells with the early T cell precursors of the human thymus. The *in vitro* generation of T cell progenitors provides a powerful model for studying the molecular mechanisms of human T cell development and improves the perspectives for T cell regenerative medicine, including chimeric antigen receptor T (CAR-T) cell therapies.

## Highlights

• We establish a protocol for generation of repopulating hPSC-derived T cell progenitors
• Transcriptome profiles of the hPSC-pro-T cells are similar to early thymic progenitors
• The hPSC-derived pro-T cells have the CD4^‒/low^CD8^‒^CD7^+^CD34^+^CD45RA^+^ phenotype
• The hPSC-pro-T cells reconstitute the thymus and develop *in vivo* into mature T cells

## Motivation

Fighting cancer and immunodeficiency disorders by adoptive transfer of T cell progenitors has certain advantages over hematopoietic stem cell transplantations (HSCTs). *De novo* generation of donor-HSC-derived T cells is slowed down at bone marrow (BM) seeding, the generation of T cell progenitors in BM, and delivery to the thymus. Lagging thymus reconstitution upon HSCT would lead to an extended period of immunodeficiency with increased health risks. Finding the source of autologous thymus-reconstituting pro-T cells would be instrumental in alleviating the T cell recovery delay upon HSCT. To address this issue, we developed a protocol for generating transplantable pro-T cells from human pluripotent stem cells.

## Graphical abstract

**Figure:**
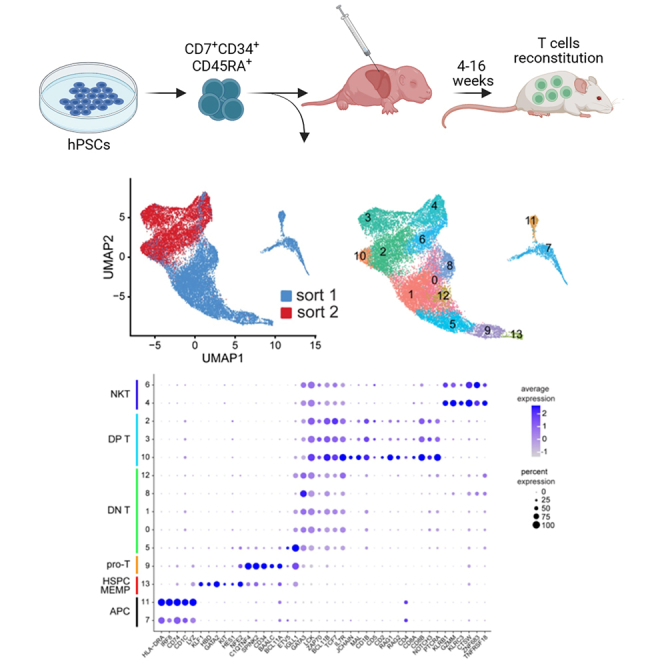

## Teaser

Philonenko et al. report generation of T cell progenitors from human pluripotent stem cells (hPSCs). These hPSC-pro-T cells demonstrate the single-cell transcription profile of early thymic progenitors from the human fetus. The T cell progenitors reconstitute the thymus over the long term and develop into mature T cells *in vivo*.

## Introduction

Human progenitor T cells (pro-T cells) are attractive tools for adoptive T cell therapies as they efficiently home to and colonize the recipient thymus, in which they proliferate and develop into mature T cells.^1^^,^^2^^,^^3^ Being immature T cell precursors, they undergo positive and negative selection in the thymus, thus becoming restricted to the host’s major histocompatibility complex (MHC). As a result, host-tolerant T lymphocytes circumvent the challenges associated with graft-versus-host disease (GVHD),^4^ which increases the chances of using off-the-shelf pro-T cells in the therapy. From a clinical perspective of fighting cancer, pathogens, and immunodeficiencies, the adoptive transfer of long-term reconstituting T cell progenitors has certain advantages over hematopoietic stem cell transplantations (HSCTs). *De novo* generation of donor-HSC-derived T cells is slowed down at bone marrow (BM) seeding, generation of T cell progenitors in BM, and delivery to the thymus.^5^ Transplantation of umbilical cord blood (UCB)-derived HSCs into conditioned immunodeficient mice resulted in high levels of BM engraftment but lacked thymus engraftment for up to 8 weeks posttransplant.^2^ In the human context, the paucity of thymus reconstitution upon HSCT would lead to an extended period of immunodeficiency with increased health risks.

An alternative approach for accelerating the recovery of the T lymphocyte compartment bypasses the need for T cell precursor delivery from the BM by adoptively transferring *in vitro*-generated pro-T cells. These T cell progenitors should be capable of immediately seeding the thymus and developing into host-tolerant, donor-derived mature T cells. Human pluripotent stem cells (hPSCs) are considered a promising source of autologous therapeutic pro-T cells due to their extensive proliferation capacity and the possibility of reliable corrective genome editing to meet specific clinical needs.

Pro-T cells that migrate, seed, and reconstitute thymuses of immunodeficient mice were successfully obtained from UCB-derived HSPCs *in vitro*.^2^^,^^3^^,^^6^^,^^7^ Generation of mouse-PSC-derived T cell progenitors with the thymus/lymphoid system reconstitution capacity was achieved by forced overexpression of *HoxB4*^8^ or *Runx1* together with *Hoxa9*.^9^ A thymus reconstitution potential was demonstrated by HSPCs generated from hPSC-derived hemogenic endothelial cells (HECs) that were lentivirally transduced by seven transcription factor genes—*ERG*, *HOXA5*, *HOXA9*, *HOXA10*, *LCOR*, *RUNX1*, and *SPI1*.^10^ This work underscores the importance of HOXA patterning of hPSC-derived mesoderm to recapitulate the HSC development. Alternative approaches for generating the thymus reconstitution potential, such as reprogramming of endothelial cells^11^^,^^12^ or mature blood cells^13^ into HSPCs, also required manipulations with the genome/epigenome by overexpression of defined transcription factors.

Unmanipulated mouse embryonic stem cell (ESC)-derived pro-T cells were capable of engrafting immunodeficient mouse recipients in the landmark work of the Zúñiga-Pflücker lab.^14^ In that study, successful homing and reconstitution of the recipients were achieved after an intermediate step of fetal thymic organ culture (FTOC) of freshly derived mouse ESC-pro-T cells. Despite the intrinsic thymus-homing defects in these T cell progenitors, the results of the study demonstrated that no genetic manipulations of PSCs were necessary for successful engraftment by PSC-pro-T cells. Unmodified hPSCs, however, have not been reported to generate progenitor T cells capable of thymus repopulation.

In this work, our efforts were focused on the derivation of thymus-repopulating cells from hPSCs without forced expression of defined factors. Employing our system of cytokine-free hematopoietic differentiation in defined conditions to produce T-cell-competent HECs, we generated hPSC-derived pro-T cells that were transcriptionally analogous to human thymocyte progenitors, efficiently reconstituted the recipient thymus, and developed into single-positive (SP) T cells within the recipient lymphoid system.

## Results

### Robust hPSC-derived T lymphopoiesis suggests the presence of a substantial progenitor population

**Figure 1:**
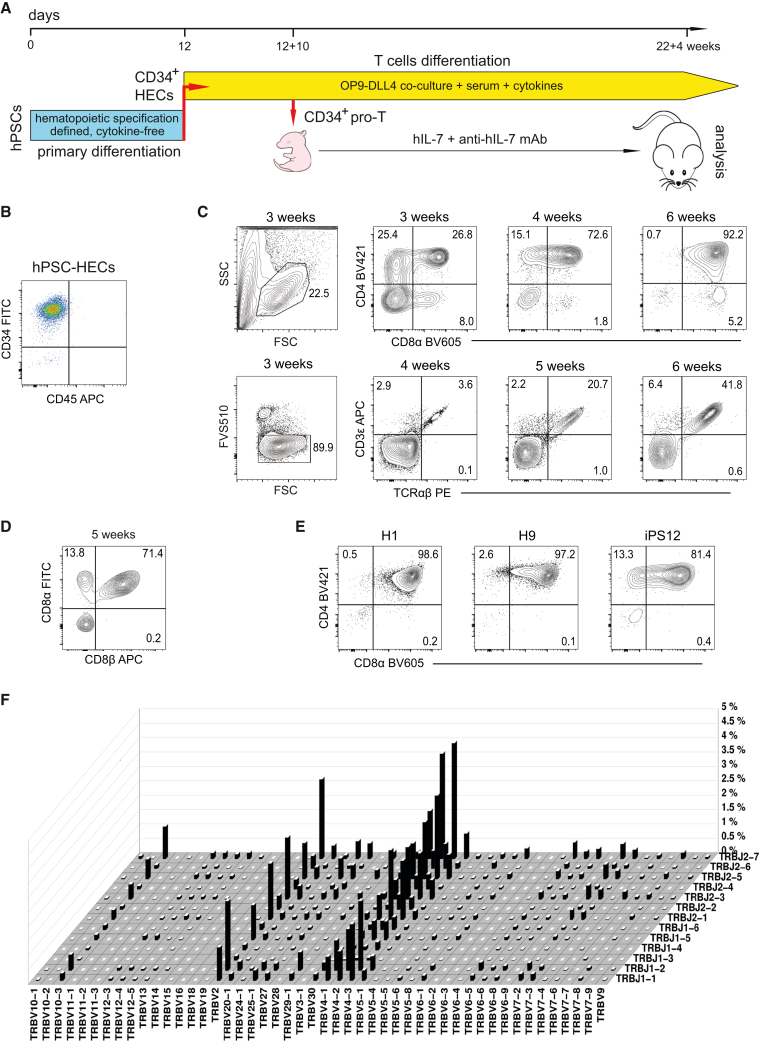
Generation of T cells from human pluripotent stem cells (A) Scheme of the two-step T cell differentiation protocol. (B) CD34^+^ HECs from SB-431542-treated day 12 primary hematopoietic differentiation cultures of H1 hESCs were used for the secondary step in the presence of OP9-DLL4 feeder cells. (C) Phenotypic dynamics of emerging T cell populations derived from CD34^+^ HECs. The gating strategy for the 3-week culture is shown as it essentially represents the strategies for all specified stages of the stromal co-culture. Here and elsewhere, representative flow cytometry data at specified time points are shown, and numbers in flow cytometry plots represent the percentages of cells within the respective quadrants. (D) Besides conventional CD8αβ^+^ T cells, a minor CD8αα^+^ population was routinely detected after 5 weeks of CD34^+^ hPSC-HEC culture on OP9-DLL4 stroma. (E) Other hPSC lines, H9 hESCs and IPS12 hiPSCs, produce lymphoid populations highly enriched in CD4^+^CD8^+^ DP T cells after 5 weeks of the co-culture. (F) TCR-seq diagram showing the spectrum and the extent of V-J rearrangements in the TCRβ locus after 7 weeks of the stromal co-culture.

As a first step toward T cells, we have employed a protocol of hPSC hematopoietic differentiation in the absence of cytokines.^15^^,^^16^ Along with many primitive progenitors, this protocol generates a large number of definitive hematopoietic precursors, including those with the T cell developmental potential. In the second phase, we cultured hPSC-derived CD34^+^ HECs (Figures 1A and 1B) on the OP9-DLL4 stroma^16^ in an optimized medium supplemented with cytokines that support T cell development. In these conditions, hPSC-HECs rapidly, within 2 weeks, differentiated into cells upregulating T cell markers, which was followed by the accumulation of CD4^+^CD8^+^ (double-positive [DP]) T cells expressing conventional CD8αβ heterodimeric co-receptor (Figures 1C and 1D). Nevertheless, a distinct CD8αα^+^ cell population was observed in the 5^th^-week co-culture, suggesting that unconventional T cells also develop in our system (Figure 1D). CD3^+^TCRαβ^+^ T lymphocytes emerged starting from the 3^rd^ week, and by the 6^th^ week, more than 40% of live cells, which were almost entirely CD4^+^CD8^+^ DP, co-expressed CD3 and TCRαβ (Figure 1C). Importantly, the co-culture conditions were suitable for efficient T cell development from several independent hPSC lines including human induced pluripotent stem cells (hiPSCs; Figure 1E). Nascent T cells intensively rearrange their *TCRβ* gene segments (Figure 1F), strongly indicating the maturation of hPSC-derived polyclonal conventional T cells. In sum, the differentiation of hPSC-HECs in the optimized co-culture conditions efficiently reproduced a robust T cell development up to the DP stage. The continuous robust proliferation of T cells indicates the presence of functional T cell progenitors in the co-culture, which prompted us to investigate whether we could identify and characterize the pro-T cell population.

### Single-cell transcriptome analysis of hPSC-HEC-derived cells

**Figure 2:**
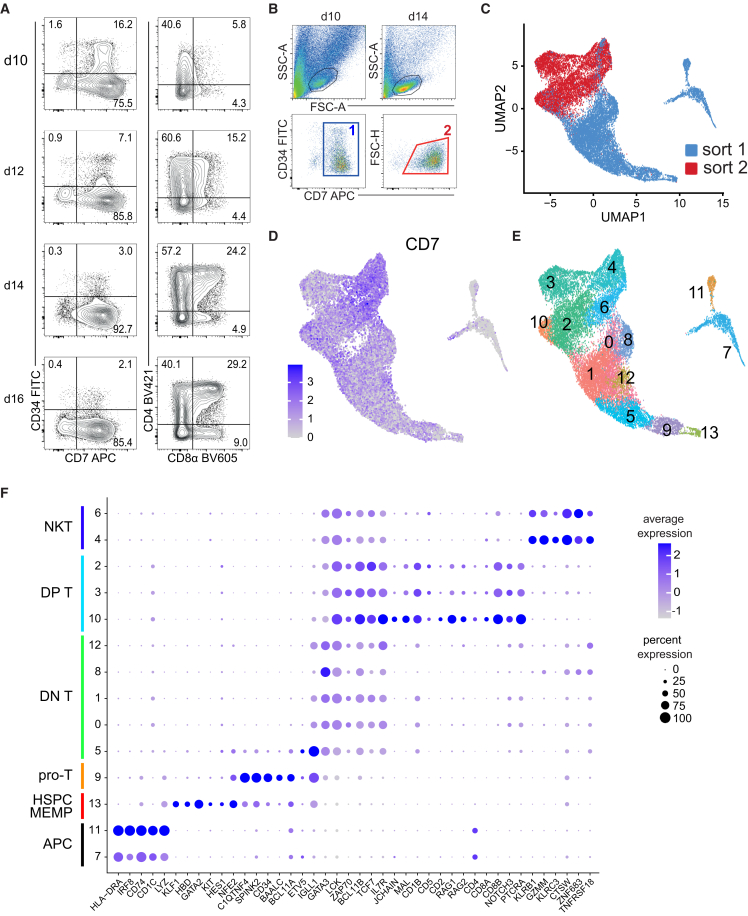
Phenotyping and scRNA-seq of the early T cells (A) Day 10–16 dynamics of H1-derived CD7^+^CD34^+^ progenitors and CD4^+^CD8^+^ DP T cells at specified time points of the lymphoid culture. (B) The sorting strategy for CD7^+^ cells at the two designated stages of the T lymphoid specification. Two upper FACS plots show the blast cell gating; corresponding sorting gates are depicted in the two lower plots. (C) Day 10 (sort 1) and day 14 (sort 2) CD7^+^ cell populations were used for scRNA-seq, and the resulting transcriptome data are visualized by UMAP. Colors highlight cells of the two sorted populations. Here and elsewhere in UMAP visualizations, each dot represents a single cell. (D) CD7 expression pattern projected on the UMAP visualization. Scaled colors represent the gene expression levels. (E) Unsupervised clustering analysis of the scRNA-seq data determined 14 cell clusters (C0–C13) marked by numbering and colors in the UMAP visualization. (F) Dot plot showing the scaled expression of selected DEGs across the CD7^+^ cell clusters. Cluster numbers on the *y* axis are as shown in (D). The feature gene expression identifies several subsets of hematopoietic and lymphoid cells annotated along the *y* axis. Scaled colors represent the gene expression levels, and the dot size encodes the proportion of positive cells in the corresponding cluster.

We looked closer into the first 2 weeks of HEC’s lymphoid development using CD34 and CD5/CD7 expression to delineate progenitors and early T cells, respectively. We found that the expression of CD34 was largely lost during the first 2 weeks of the OP9-DLL4 co-culture, while CD7^+^/CD5^+^CD34^‒^ cells continued to dominate the human domain of the co-culture (Figures 2A and S1). CD4^+^CD8^+^ DP T cells emerged starting from days 10–12, heralding the transition toward the T cell maturation (Figure 2A).

To identify putative ETPs, we sorted CD7^+^ cells from day 10 and day 14 of the co-culture (Figure 2B) and subjected them to single-cell RNA sequencing (scRNA-seq). These stages delineate a period between the mass emergence of HEC-derived hematopoietic cells and a strong decline of CD34 expression in these cells, which indicates the depletion of progenitors. Before day 10, the total number of hematopoietic cells was too low for further studies. Overall, we sequenced the transcriptome of 12,072 day 10 cells, 3,273 genes per cell on average, and 8,793 day 14 cells, 3,042 genes per cell at average. The two datasets were aggregated and visualized using the UMAP algorithm. The visualization showed the radical progress of lymphopoiesis between day 10 and day 14 as the transcription profiles of these two stages changed dramatically with minimal overlap (Figure 2C). A majority of cells from both stages expressed several key T cell regulatory genes, such as *LAT*, *LCK*, *IL7R*, *GATA3*, *BCL11B*, *LEF1*, *TCF7*, and *ZAP70* (Figure S1B). In contrast, mRNAs of the identity T cell markers, PTCRA, CD8β, TRAC, CD8α, and CD4, were detected mainly in the day 14 zone. Similar to *in vivo* lymphopoiesis, *PTCRA* (pre-T cell antigen receptor α) and *TRAC* (T cell receptor α constant) expression patterns were mutually exclusive, identifying two separate populations of pre-T and early T cells, respectively. Overall, during the specification of the CD7^+^ T lineage, three major overlapping differentiation stages can be outlined: *CD34*^+^ progenitors, *CD7*^+^/*CD3ε*^+^ nascent T cell lineage, and further differentiated *RAG1*^+^/*RAG2*^+^/*CD4*^+^*CD8β*^+^ cells (Figure S2).

Unsupervised clustering of the sequencing data recognized 14 CD7^+^ cell clusters, C0–C13, with distinct gene expre**s**sion profiles (Figures 2D, 2E, and S3). We selected several dozen differently expressed genes (DEGs, log_2_FC [fold change] > 0.5) to define and characterize the clusters and applied a descriptive annotation based on the expression of feature genes (Figure 2F). To identify potential markers for the FACS enrichment of putative ETPs, we investigated the transcription profile of DEGs encoding cell surface proteins throughout the annotated clusters. The expression pattern of these genes was generally consistent with the cluster annotation (Figure S4).

### Parsing transcriptional DN and DP stages of hPSC-HEC-T cells

**Figure 3:**
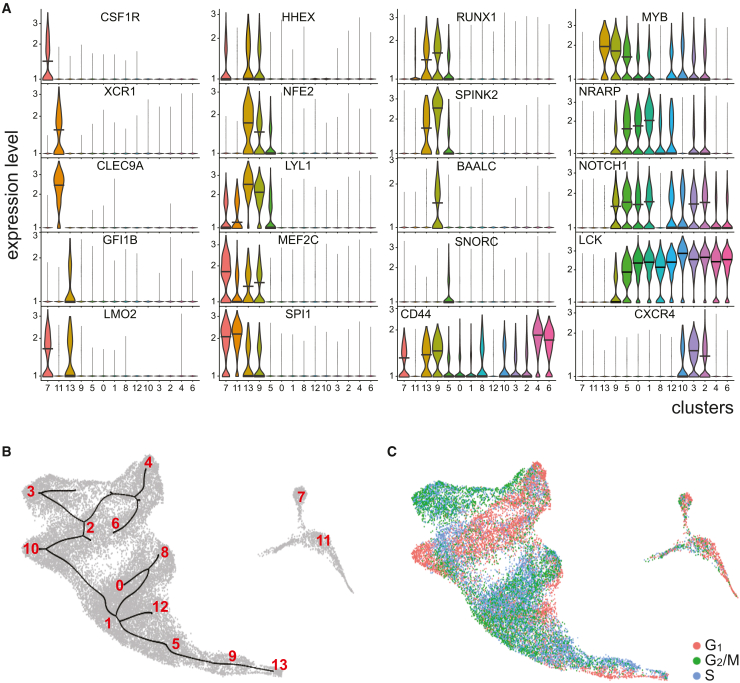
Characterization of CD7^+^ cells generated by the hPSC differentiation (A) Violin plots showing the expression of feature genes in the CD7^+^ cell clusters. Here and elsewhere, median expression levels are shown by horizontal bars. (B) A pseudotime trajectory projected on the UMAP visualization shows a distinct developmental pathway among CD7^+^ cells. The cell cluster regions are designated by red numbers. (C) Profiling of cell-cycle gene expression across the hPSC-HEC-derived clusters. Dot colors designate the phases of the cell cycle assigned to individual cells.

*GATA3/LCK/IL7R/BCL11B/TCF7 (GLIBT)*-positive cluster group consisting of C0, C1, C5, C8, and C12 did not express *CD4* and *CD8* (Figure 2F), suggesting that it represents an earlier stage of T cell development. In contrast, *GLIBT*-positive clusters 2, 3, and 10 contained cells that were more advanced in the T lineage specification, being positive for *CD4* and *CD8* transcripts (Figure 2F) and upregulating *CD1B*, *IL7R*, *IL2RG*, *PTPRC* (*CD45*), and *TRBC1* (Figure S4). These transcriptional double-negative (DN)- and DP-like clusters were annotated as DN T and DP T, respectively (Figure 2F). Among the DN T cluster group, C5 was distinguished by markedly deep and intensive expression of *IGLL1* (Figure 2F), а functional marker of pre-B cells and human HSPCs,^17^ an observation suggesting that the cluster represents a subset of early lymphoid progenitors. In contrast to other DN T clusters, C5 expressed, although at relatively low levels, genes encoding key hematopoietic transcription factors NFE2, LYL1, RUNX1, and another marker of HSPCs, SPINK2 (Figure 3A). The pseudotime trajectory analysis predicted C5 as the most proximal among the DN cells (Figure 3B), suggesting that it represents the earliest T-lineage-committed cells. In the DP T cluster group, C10 expressed higher levels of *PTCRA*, *RAG1,* and *RAG2* in a substantially larger subset of cells compared to C2 and C3 (Figure 2F), indicating that C10 cells were more immature. All clusters of the group displayed an overwhelming excess of *CD8B* transcripts over those of *CD8A* and *CD4*. The expression profiles of feature genes were remarkably similar across three DP T clusters (Figures 2F and S4) and differed largely by the expression of cell cycle genes (Figure 3C). The observed transcriptional variations among hPSC-derived DN and DP T cell clusters evoke a comparison with the heterogeneity of early T cell populations in the thymus.^18^

### Other thymocyte lineages were detected in the hPSC-derived lymphopoiesis

Closely related and largely composed of day 10 cells, *GLIBT*-negative C7 and C11 are located off the T-lymphoid “tree” on the UMAP visualization (Figures 2C and 2D). These clusters were prominent in their robust and broad expression of MHC class II HLA transcripts, *DRA*, *DRB1*, *DPA1*, *DQB1*, and *DQA1* (Figure S4), and thus were annotated as antigen-presenting cells (APCs). Of note, a larger proportion of C11 expressed higher levels of these transcripts compared to C7. Moreover, C11 cells transcribed significantly higher levels of DC-specific *DNASE1L3* and *CADM1* (Figures S3 and S4), as well as *IRF8* (Figure 2F), which is preferentially expressed in DCs within the myeloid compartment.^19^ Furthermore, *XCR* and *CLEC9A*, the identifiers of the DC lineage, were specifically expressed in C11 and the monocyte marker *CSF1R* in C7 (Figure 3C). Therefore, we identified C11 as of dendritic cell lineage, while C7 encompassed cells committed toward monocytes/macrophages.

Within the T-lymphoid transcriptome mainstream, C4 and C6 selectively and nearly ubiquitously expressed the markers of the natural killer T cell (NKT) lineage, such as *CXCR3*, *KLRB1*, and *CLEC2D* (Figures 2F and S4). Consistent with the NKT cell identity, C4 and C6 prominently expressed several other NKT feature genes as well as genes that are specifically upregulated in both T and NK cell lineages: *LCK*, *GATA3*, *ZAP70*, *KLRB1*, *CD96*, *GZMM*, *TNFRSF18*, and *ZNF683* (Figure 2F).^19^ Practically all cells in these clusters expressed mRNAs of diagnostic T cell markers CD3D and CD3G at high levels, which, in combination with the expression of the abovementioned genes, strongly indicates the NKT identity of C4 and C6. A pseudotime trajectory (Figure 3B) suggested an ontogenic connection between the DP clusters (C2, C3, and C10) and NKT cells, thus recapitulating the thymic process of NKT cell development.^20^

### Identifying T cell progenitors

At the start of the pseudotime trajectory (Figure 3B), *CD34*^low^ Cluster 13 exhibited a signature of myeloid and erythro-megakaryocyte progenitors specifically expressing *CD33*, *GATA2*, *NFE2*, *HBD*, *KLF1*, *KIT (CD117)*, and *TFRC (CD71)* (Figures 2F and S4). Cells of the cluster also strongly and broadly upregulated genes encoding tetraspanins CD63 and CD82, sialomucin CD164, and integrin ITGA4 (Figure S4), the functional markers of human HSPCs.^21^^,^^22^^,^^23^ Furthermore, a large proportion of C13 preferentially expressed several hematopoietic transcription factor genes, such as *GFI1B*, *MYB*, *LYL1*, *LMO2*, *HHEX*, and *RUNX1* (Figure 3A). Conversely, the marker genes of lymphoid differentiation, *CD2*, *CD4*, *CD5*, *CD8A*, *CD8B*, *RAG1*, *RAG2*, *JCHAIN*, and others, were strongly repressed (Figure 2F). The above observations suggested that cluster 13 represents myeloid and megakaryocyte/erythroid specification of HECs, leading to the emergence of precursors resembling megakaryocyte-erythroid-mast cell progenitors (MEMPs).^24^

In contrast to C13, the adjacent C9 exhibited a strong, broad, and nearly selective expression of *CD34*. С9 shared with C13 the expression of several hematopoietic transcription factors, *RUNX1, MYB, LYL1*, and *MEF2C*, whereas C9 cells uniquely upregulated *BAALC* (Figure 3A), a marker of CD34^+^/CD133^+^ HSPCs.^17^^,^^25^ Along with a strong expression of other HSPC markers, *BCL11A*, *SPINK2*, and *C1QTNF4*,^17^ nearly all cells of C9 upregulated *SELL* (*L-selectin*) (Figure S4), encoding a lymph node and thymus trafficking molecule expressed in lymphoid-primed HSCs.^26^ Moreover, while downregulating *TFRC*, *KLF1*, *GATA2*, *HBD*, and *NFE2*, C9 expressed T-lymphoid genes, *LCK*, *GATA3*, *NOTCH1*, *NRARP*, *CD3D*, *IL2RG*, *BCL11B*, and *CD3G*, at higher levels and more broadly compared to C13 (Figures 2F, 3A, and S4). Taken together, these observations suggest that C9 is comprised of early progenitors that commit toward the T-lymphoid lineage.

C9 cells were only partially proliferative, while the adjacent downstream DN T cells of C5 were actively engaged in cell division (Figure 3C). This observation indicated that C9 cells could hardly recapitulate thymus-resident ETPs, which are strongly proliferative.^27^ The high levels of *CD34*, *SELL*, *ITGAE*, and *ITGA4* transcripts suggest that C9 contains migratory precursors of these ETPs. To clarify this issue, we compared the transcriptomes of the hPSC-HEC-CD7^+^ cells and human thymocytes.

### Many hPSC-HEC-T cells are transcriptionally similar to human thymocytes

**Figure 4:**
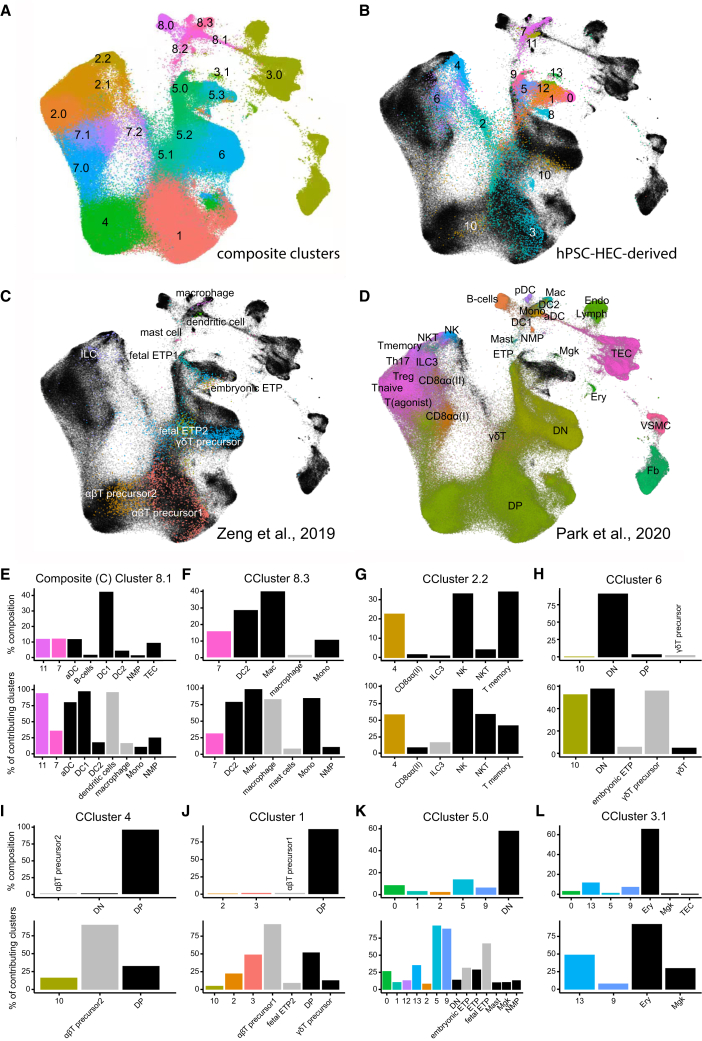
Matching up the expression profiles of hPSC-HEC-CD7^+^ cells to reference data from human thymocyte populations (A) Unsupervised clustering of the integrated scRNA-seq data from three sources: two studies of human thymocytes^28^^,^^29^ and the sequencing of the hPSC-HEC-derived cells. Numbers and colors mark the individual composite clusters (CCs). (B) Highlighting gene expression profiles of the hPSC-HEC-CD7^+^ clusters, differentially colored, in the combined three-source UMAP visualization, in black. (C) Mapping Zeng et al.^28^ data, colored, on the combined three-source UMAP visualization, in black. Cell clusters are labeled as indicated in Zeng et al.^28^; ILC, innate lymphoid cell; ETP, early thymic progenitor. (D) Mapping Park et al.^29^ data, colored, on the combined three-source UMAP visualization, in black. Cell clusters indicated are DN and DP T cells. aDC, activated dendritic cells; DC1 and DC2, conventional dendritic cells 1 and 2; pDC, plasmacytoid dendritic cells; NMP, neutrophil-myeloid progenitor; Mono, monocyte; Mac, macrophage; Mgk, megakaryocyte; Endo, endothelial cells; VSMC, vascular smooth muscle cells; Fb, fibroblasts; Ery, erythrocytes; TEC, thymic epithelial cells; Lymph, lymph nodes. (E) Composition of CC8.1 and strength of thymocyte and hPSC-HEC-derived cluster contribution into the composite cluster. The location of all composite clusters is shown in (A). Here and in (F)–(L), the upper bar plot shows the percentage contribution of the source clusters to the designated composite cluster. The bottom plot shows the percentage of cells in source clusters contributing to the composite cluster. Colored bars represent the hPSC-HEC-derived cell clusters, black bars Park et al.^29^ clusters, and gray bars Zeng et al.^28^ clusters. (F–L) Composition of the designated CC clusters and the strength of the source cluster contribution to the CCs.

We integrated our single-cell gene expression data with the published scRNA-seq profiles of human thymocytes at various stages of development.^28^^,^^29^ We constructed a mixed transcriptome landscape using the Conos software^30^ and re-clustered the composite data (Figures 4A–4D). To infer the identity of our cell populations and their similarity with the *in vivo* cells, we measured (1) the component percentage of the composite clusters and (2) fractions of the *in vitro* and the thymocyte clusters that contribute to each composite cluster. Cell frequencies in the original *in vivo* and *in vitro* clusters can vary significantly, and the second measurement can be considered a normalization of the composite cluster structure. The approach can verify the annotation of the hPSC-HEC-CD7^+^ clusters (C0–13) and helps to assess their heterogeneity.

As shown in Figures 4 and S5, a majority of composite clusters contained hPSC-HEC-derived cells together with various populations of thymocytes. Only a few composite clusters excluded either the hPSC-derived populations or the thymocytes (Figure S5), suggesting a good phenotype correlation between the thymocytes and the *in vitro*-generated cells. In particular, C7 and C11 formed the composite cluster 8.1 (CC8.1) together with thymic cells dominated by a subtype of DCs (Figure 4E); 75%–100% of C11, activated DC, and DC1 of Park et al. and dendritic cells of Zeng et al. contributed to CC8.1, which indicated a relative homogeneity and strongly confirmed the identity of C11. The composite cluster approach also confirmed predominantly monocyte/macrophage identity of C7 (Figure 4F). The NKT identity of C4 was similarly demonstrated by the strong resemblance of its transcription profile to NK and NKT cells of Park et al. (Figure 4G). Thus, the composite clustering analysis confirmed that the hPSC-derived cells were transcriptionally similar to thymic DCs, macrophages, and NKT cells.

About 50% of C10, along with γδT precursors and DN thymocytes, contributed to CC6, 15% of C10 together with αβT precursors and DP cells to CC4, and 5% of the cluster to CC1, dominated by αβT precursors and DP cells (Figures 4H–4J). This profile is largely consistent with the attribution of C10 as the most immature DP T cells.

Notably, almost the entire C5 and C9 preferentially shared CC5.0 with 67% of fetal ETPs of Zeng et al. (Figure 4K). Moreover, significant (>25%) fractions of C13, ETPs of Park et al., and embryonic ETP of Zeng et al. also contributed to CC5.0. These findings indicate that C5, C9, and C13, the only clusters that expressed CD34 (Figure S3), displayed the transcriptional signature of human ETPs. These three clusters were included in the erythro-megakaryocytic CC3.1, but only around 8% of C9 and less than 1% of C5 (below the threshold) contributed to that composite cluster, in which almost 50% of C13 was clustered together with 100% erythrocytes and over 25% megakaryocytes of Park et al. (Figure 4L). The observation confirmed that C13 largely consists of the erythro-megakaryocyte progenitors. The similarity of C9 cells to fetal and embryonic ETPs is in line with the notion that they represent the migratory precursors of ETPs, the thymus seeding progenitors (TSPs).^31^^,^^32^ Taken together, the abovementioned data demonstrate that the differentiation of hPSC-HECs generates a number of thymocyte-like cell populations, including the analogs of ETP/TSP cells.

### Human PSC-HEC-derived *CD34*^+^ pro-T cells express T lymphocyte homing genes

**Figure 5:**
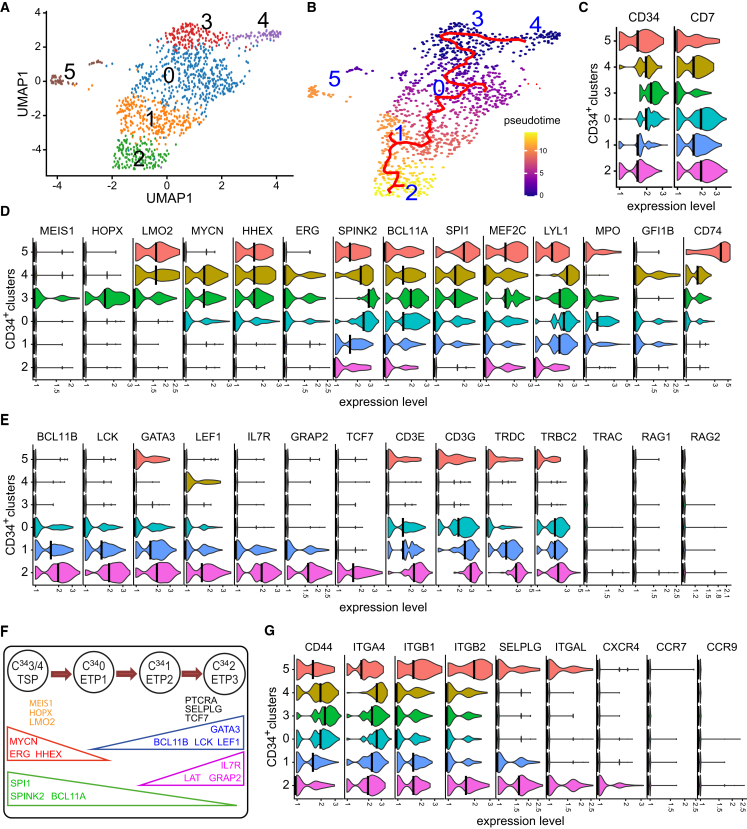
Transcription profiling of *CD34*^+^ progenitors generated from hPSC-HECs (A) The UMAP visualization and unsupervised clustering analysis of the *CD34*-positive domain within the СD7^+^ transcriptome. The analysis determined six *CD34*^+^ cell clusters marked by numbers and colors. (B) A pseudotime trajectory projected on the UMAP visualization shows a distinct developmental pathway throughout the *CD34*^+^ cell clusters. (C) Violin plots showing the expression profile of *CD7* and *CD34* across C^34^ clusters. (D) Gene expression of key hematopoietic transcription factors and CD74 across C^34^ clusters. (E) Expression of feature T lineage genes in the *CD34*^+^ clusters. (F) A model showing the development of TSP/ETP-like progenitors represented by C^34^0-4 clusters. (G) Violin plots showing the gene expression of key homing molecules by C^34^ clusters.

Next, we looked deeper into the transcription profile of hPSC-HEC-derived cells that express *CD34*. Unsupervised re-clustering of the *CD34*^+^ cells (with normalized *CD34* expression >0.1; Figure S6A) led to the recognition of six distinct cell clusters (Figures 5A and 5B). CD7^+^ C9 was represented broadly (57.6%) in the *CD34*^+^ clusters, while almost the entire CD7^+^ C13 contributed exclusively to *CD34*^+^ cluster 4 (C^34^4) (Figure S6B). Except for C^34^5, which co-localized with the CD7^+^ APC cells (Figure S6C) and thus represented a myeloid direction of *CD34*^+^ progenitor specification, C^34^0–C^34^3 and a few cells from C^34^4 could be joined by a single pseudotime trajectory, suggesting the developmental interconnection between them (Figure 5B). C^34^3 represented the most primitive T lineage cells, distinguished by the highest expression of *CD34* and the lowest level of CD7 mRNA (Figures 5C, S6D, and S6E). These cells, along with cells of C^34^4, preferentially upregulated a number of HSPC transcription factor genes, such as *MEIS1*, *HOPX*, *LMO2*, *MYCN*, *HHEX*, *ERG*, *SPINK2*, *BCL11A*, *SPI1*, *MEF2C*, and *LYL1*, and generally lacked the transcripts encoding T-cell-specific molecules, GLIBT, LEF1, GRAP2, LAT, ZAP70, CD3E, CD3G, TRGC1, TRDC, TRBC2, PTCRA, TRAC, RAG1, and RAG2 (Figures 5D and 5E). In contrast, most of these functional T cell markers became gradually upregulated in more differentiated, downstream clusters C^34^0, C^34^1, and C^34^2 (Figure 5E). Correspondingly, many of the HSPC-related transcription factor genes were progressively repressed in the downstream clusters (Figures 5D and 5F).

We then analyzed the expression of thymus homing and seeding molecules. *CD44* and *ITGA4*, the HSPC markers mediating the adherence to the thymus endothelium,^33^ were strongly and broadly expressed across all C^34^ clusters (Figure 5G). Genes encoding integrins, ITGB1, ITGB2, ITGAL, and a counter-receptor of selectins, SELPLG, were upregulated in the downstream clusters C^34^0, 1, 2, and in the myeloid C^34^5. The key homing molecules CXCR4, CCR7, and CCR9 also showed a tendency to upregulation in the downstream clusters. Moreover, these clusters strongly expressed *BCL11B* (Figure 5E), the key transcriptional inducer of CCR7 and CCR9 receptors, which direct the movement of progenitors from bone marrow to the thymus. In sum, the reclustering data indicate that hPSC-HECs produced *CD34*^+^ progenitors with a thymus homing and seeding potential, which qualifies them as TSP-like cells.

### hPSCs-HEC-derived T cell progenitors initiate T-lymphopoiesis *in vivo*

**Figure 6:**
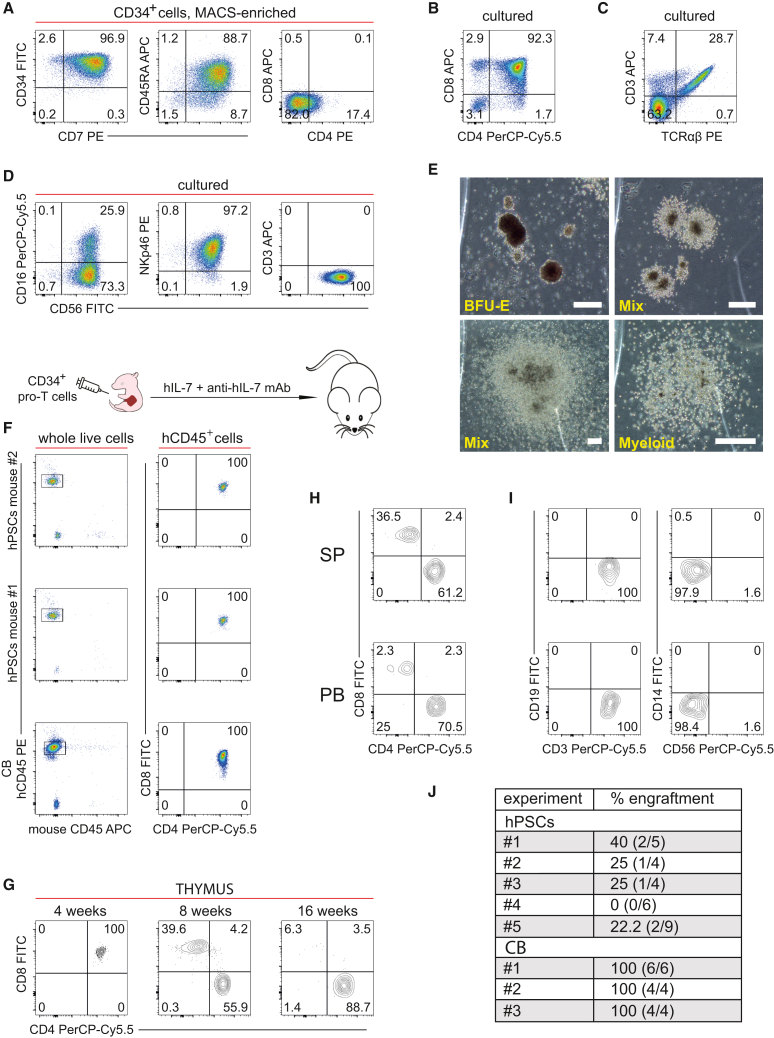
The *in vitro* and *in vivo* potential of hPSC-HEC-derived CD34^+^ cells (A) Phenotyping of MACS-purified CD34^+^cells from day 10 co-culture. (B) CD4/CD8 profiling of hPSC-HEC-CD34^+^ cells cultured for 3 weeks in the T-cell-promoting conditions on OP9-DLL4 stroma. (C) CD3/TCRαβ flow cytometry analysis of the same cells as in (B). (D) Flow cytometry of hPSC-HEC-CD34^+^ cells cultured for 3 weeks in the NK cell-promoting conditions on OP9-DLL4 stroma. (E) In an assay medium supplemented by hematopoietic cytokines, a small proportion of hPSC-HEC-CD34^+^ cells produce erythroid, myeloid, and mixed colonies. BFU-E, burst-forming units erythroid; CFU-Myeloid, colony-forming units-myeloid; CFU-Mix, colony-forming units mixed erythro-myeloid colonies. Scale bars, 200 μm. (F) Thymus engraftment by hPSC-HEC-CD34^+^ cells and cultured UCB-CD34^+^ progenitors in 4-week-old mice. Four top FACS plots show the flow cytometry analysis of hCD45^+^ cells isolated from two thymuses that were repopulated by hPSC-HEC-derived pro-T cells. The plots below show the results of the reference transplantation by UCB-derived CD34^+^progenitors that were cultured in the same conditions for 10 days. (G) Long-term thymus repopulation by the hPSC-HEC-derived TSPs. Human CD45-positive DP T cells of 4-week-old mice differentiated into SP T cells at 8 weeks posttransplant, and by 16 weeks, the majority of human T lymphocytes in the thymus had the CD4^+^CD8^─^ phenotype. (H) The progeny of thymus-rescuing hPSC-HEC-derived TSPs was capable of migrating into the peripheral blood (PB) and spleen (SP) of the recipient mice. Flow cytometry analysis of human CD45-positive PB and SP cells 8 weeks after the transplantation is shown. (I) Engraftment potential of hPSC-HEC-derived TSPs is limited to the T cell lineage. No hCD14^+^ monocytes/macrophages, hCD19^+^ B cells, and hCD56^+^ NK cells were detected 8 weeks posttransplant. (J) The thymus repopulation efficiency of hPSC-HEC- and CB-derived progenitors cultured for 10 days on OP9-DLL4 stroma. The percentage of repopulated animals for each transplantation experiment is shown in the table; in parentheses, the number of repopulated/transplanted animals is shown. Recipient mice were considered repopulated if human T cells persisted in the thymus for more than 4 weeks after transplantation.

We then examined whether the transcription profile of the TSP-like cells translates into a functional potential. First, we assessed the *in vitro* lymphoid and erythro-myeloid potential of the putative TSPs. Almost all freshly sorted day 10 hPSC-HEC-CD34^+^ cells co-expressed CD7, and nearly 90% of the cells were positive for CD45RA, a marker of multi-lymphoid progenitors within the CD34^+^ cell population (Figure 6A).^27^ CD8 and CD4 were either missing or expressed at a low level, respectively (Figure 6A). Low levels of CD4 expression have been indicated as a characteristic feature of mouse ETPs/TSPs.^34^

We then subjected the CD34^+^ cells to further co-culturing with OP9-DLL4 stroma and a clonogenic progenitor assay. In the 3-week co-culture, hPSC-HEC-CD34^+^ progenitors generated a lymphocyte population that consisted almost entirely of DP T cells with a minor contribution of SP T cells (Figure 6B). Nearly 30% of the hPSC-HEC-CD34^+^ progeny co-expressed CD3 and TCRαβ, manifesting T cell receptor complex on the cell surface (Figure 6C). When interleukin 15 (IL-15) was added to the co-culture, hPSC-HEC-CD34^+^ progenitors differentiated into CD3^‒^CD56^+^NKp46^+^ conventional NK cells that partially expressed CD16 (Figure 6D). In the clonogenic assay, a small (1–2%) fraction of the CD34^+^ progenitors gave rise to erythro-myeloid cell colonies (Figure 6E). Altogether, these results demonstrated that the CD34^+^ population contained early lymphoid precursor cells along with a few erythro-myeloid progenitors that were identified in partially *CD34*-positive CD7^+^ cluster 13.

To investigate the functional potential of the hPSC-HEC-derived lymphoid progenitors *in vivo*, we injected day 10 hPSC-HEC-CD34^+^ cells into the liver of non-conditioned newborn NSG-type mice (NSI mice)^35^ that were administered periodic doses of human IL-7 together with anti-hIL-7 antibodies. We found that these cells successfully engrafted and reconstituted the thymuses of the recipient mice at 4, 8, and 16 weeks post-transplantation. Two representative experiments shown in Figure 6F demonstrated that reconstituted thymuses in 4-week-old mice were comprised of human CD45^+^CD4^+^CD8^+^ DP T cells. Comparable transplantation results were obtained with CD34^+^cells isolated after a 10-day culture of CD34^+^ UCB cells on OP9-DLL4 stroma in the same conditions (Figure 6F).

The transplanted cells apparently entered the canonical T cell development pathway in the reconstituted thymus: first from injected DN CD34^+^into DP T cells at 4 weeks posttransplant and then, by 8 weeks, into CD4^+^CD8^‒^ and CD4^‒^CD8^+^ SP T cells (Figure 6G). At 16 weeks, the great majority of human thymocytes had the CD4^+^CD8^‒^ SP phenotype. In contrast, human CD4^+^CD8^+^ DP T cells were still present in the thymuses of mice injected with the UCB-derived progenitors at 16 weeks post-transplant (Figure S7A). hPSC-derived CD45^+^CD8^+^ and CD45^+^CD4^+^ SP T cells were observed in the spleen and peripheral blood of recipients 8 weeks after transplantation (Figure 6H). The hPSC-HEC-CD34^+^ cells failed to contribute to B, NK, and myeloid lineages in the spleen and blood, as indicated by the absence of CD19, CD56, and CD14 expression, respectively (Figure 6I). In contrast, although no myeloid and NK cell progeny of the UCB-CD34^+^ cells were detected in the spleen, blood, and thymus (Figures S7B and S7C), these cultured progenitors developed into CD3^‒^CD19^+^ B cells that repopulate the spleen and peripheral blood of transplanted mice (Figure S7B). These experiments showed that our culture conditions supported the development and/or survival of human B cell progenitors. Thus, the failure of the B cell engraftment by the hPSC-HEC-CD34^+^cells suggested that the lymphoid developmental potential of these progenitors was more restricted compared to the UCB-CD34^+^population. Altogether, the *in vitro* and *in vivo* potential studies demonstrated that hPSC-HECs generated the thymus repopulating pre-T cells that have the CD4^‒/low^CD8^‒^CD7^+^CD34^+^CD45RA^+^ phenotype.

In summary, we have shown that hPSC-HEC-derived CD34^+^ cells have the ability to home to the thymus, rescue and repopulate it, and differentiate into DP and then SP T cells that can leave the thymus and migrate to peripheral tissues. The presence of such thymus-reconstituting progenitors was predicted by the single-cell transcriptome analysis. The efficiency of thymus reconstitution by the hPSC-TSPs varied from 0% to 40% in five independent transplantation experiments with an average of 22.44%, while CD34^+^ UCB cells reconstituted thymuses of all transplanted mice (Figure 6J). Nevertheless, achieving thymus reconstitution with hPSC-derived cells provides a basis for further technological improvement of hPSC-HEC-TSP-like cell generation.

## Discussion

In this study, we generated hPSC-derived T cell progenitors capable of long-term T lymphopoiesis *in vivo* without forced expression of defined transcription factors. These cells display characteristics of thymus-seeding progenitors and restore the function of the thymus. Our proof-of-principle research provides a step toward efficient autologous T cell transfer immunotherapy for cancer. In this therapy, pro-T cells would have a significant advantage over mature T cells since they can replenish the pool of exogenous T lymphocytes *in vivo*. Armed with CARs, the progenitors can ensure the prolonged therapeutic effect, guarding against cancer recurrence and setting up conditions to fight solid tumors. Furthermore, the generation of genetically unmodified T cell progenitors in recapitulative culture conditions creates a reliable model for studying the molecular mechanisms of T cell development in humans. In addition, with the derivation of patient-derived hiPSC lines, the model can be employed for studying T cell involvement in the development of pathological conditions such as SLE.^36^

Unmodified human PS cells were recently successfully used as a source of HSCs endowed with long-term high-level multilineage engraftment potential.^37^ The study demonstrates that proper conditioning of differentiating hPSCs leads to the emergence of long-term engraftment potential. Making such unmodified HSCs in a dish has major clinical ramifications. However, for cancer immunotherapy, T cell progenitors, especially those that are capable of long-term engraftment, have a critical advantage over HSCs due to their direct boost to the immune system. Moreover, co-transplanted with HSCs, pro-T cells facilitate HSC-derived thymic engraftment, helping to overcome the paucity of T cell reconstitution after HSCT.^2^^,^^3^

hPSC-derived HECs were previously shown to contain pro-T cells capable of generating T lymphocytes *in vitro*.^38^ To produce pro-T cells, endowed with an *in vivo* repopulation potential, we have employed a two-stage hPSC differentiation protocol promoting a robust expansion of CD34^+^ HECs that initiate extensive T cell development on OP9-DLL4 in the presence of lymphoid cytokines. In addition to T and NKT lineages, we observed the specification of АРСs, which, according to a previous report,^39^ might be initiated by the increased Notch signaling. The inefficient maturation of hPSC-HEC-derived pro-T cells into SP T cells (Figure 1C) was apparently due to the absence of a thymic environment in our cell cultures. After being introduced into the newborn liver, these progenitors migrated to the thymus and eventually developed there into SP T cells.

Single-cell gene expression analysis of hPSC-HEC-derived cells and their comparison with human thymocytes showed CD7^+^ cluster 9 containing cells that are closely related to human ETP/TSP-like cells on the transcription level. The C9 cells preferentially express а number of feature HSC genes, as well as markers of the T cell lineage. Testing the thymus rescue potential of the CD34^+^ population, which largely consisted of C9 cells, demonstrated that the repopulation-competent hPSC-derived cells lacked the long-term repopulation capacity of HSCs. These observations confirmed the identity of the generated TSP-like cells as progenitors possessing a more restricted self-renewal potential compared to HSCs. Overall, the most impressive conclusion drawn from the single-cell analysis was that the *in vitro* model system paralleled the *in vivo* T cell development, producing populations with expression signatures similar to those of fetal and postnatal thymocytes. These findings add a significant argument for the utility of the hPSC differentiation approach in clinical research.

It is reasonable to assume that the quality of the hPSC-derived HEC population was the key to the successful generation of thymus-reconstituting early T cell progenitors. The method of planar cytokine-free hPSC differentiation in defined conditions closely recapitulates early human hematopoietic development.^15^ Accordingly, HECs that were generated by the method resemble their *in vivo* counterparts, being capable of producing cells analogous to TSPs.

It is thought that hematopoietic differentiation of hPSCs reproduces the yolk sac hematopoiesis,^40^^,^^41^ and the Notch-signaling-driven differentiation of hPSCs recapitulates T lymphopoiesis that emerges in the human and murine conceptuses ahead of and independently of adult hematopoietic hierarchy.^42^^,^^43^^,^^44^^,^^45^^,^^46^^,^^47^ Lympho-myeloid progenitors rather than HSCs have been shown to initiate thymopoiesis in the embryo.^44^ Thymus engrafting hPSC-TSP-like cells may recapitulate T-lineage-restricted derivatives of the yolk sac-type lymphoid-primed multipotent progenitors (LMPPs).^46^^,^^48^ The expression of HSC genes by these cells is not surprising, according to an observation that some early yolk sac progenitors and HSCs represent the same cell lineage.^49^

### Limitations of the study

In our xenogeneic transplantation experiments, we used immunodeficient mice that lacked all types of lymphocytes. Cancer patients who can hardly be subjected to lymphoablation would offer a lot more competitive environment to hPSC-HEC-TSPs. However, upon prospective transplantations into patients, the TSPs will be exposed to the autologous lymphopoietic microenvironment, which would strongly support their survival and development. Besides, potentially unlimited numbers of hPSC-derived cells could increase the engraftment efficiency in unconditioned recipients. Another issue is that the newborn transplantation format cannot be used in cancer treatment. So far, all our attempts to transplant hPSC-HEC-CD34^+^ cells into adult recipients have not led to any detectable engraftment. Moreover, we demonstrated a lower thymus-rescuing potential of hPSC-HEC-CD34^+^ cells compared to the cultured CB-CD34^+^ cells. These data suggest that hPSC-HEC-TSPs require further adaptation to the fetal lymphopoietic microenvironment. We, therefore, consider an additional organoid co-culture with fetal thymus or liver cells to properly “educate” the hPSC-HEC-TSP for efficient engraftment of adult recipients. Overcoming the current limitations will provide a foundation for further development of strategies aimed at the generation of therapeutic pro-T cells.

## Resource availability

### Lead contact

All requests should be directed to the lead contact, Igor M. Samokhvalov (igormikhail@aol.com).

### Materials availability

This study did not generate new unique reagents.

### Data and code availability

• The scRNA-sequencing data are available from NCBI GEO with the accession number GSE202253.
• This paper does not report the original code.
• Any additional information required to reanalyze the data reported in the paper is available from the lead contact upon request.

## Acknowledgments

This work was supported by the 10.13039/501100012166National Key R&D Program of China (2017YFA0103101), the 10.13039/501100012245Science and Technology Planning Project of Guangdong Province, China (2017B030314056 and 2020B1212060052), the 10.13039/501100012166National Basic Research Program of China (2015CB964900), the Guangdong Province Leading Talent Program 2014–2018 (to I.M.S.), CAS President’s International Fellowship Initiative (PIFI) visiting fellow funding 2015–2017 (to E.S.P.), Russian Science Foundation (grant no. 23-64-00002) (to P.V.), and the Ministry of Science and Higher Education of the Russian Federation (project # FGFG-2025-0017) (to D.M. and E.A.).

## Author contributions

Conceptualization, E.S.P., B.Z., I.M.S., and P.V.; methodology, E.S.P., B.Z., Z.S., P.L., Y.S., D.M., and E.A.; investigation, E.S.P., B.Z., E.A., Z.S., and I.M.S.; visualization, E.S.P., B.Z., Z.S., and E.A.; data curation, E.A. and Z.S.; funding acquisition, P.L., P.V., and I.M.S.; project administration, E.S.P., P.L., and I.M.S.; writing—original draft, I.M.S.; writing—review & editing, E.A., D.M., and I.M.S.; supervision, P.V. and I.M.S.

## Declaration of interests

The authors declare that they have no conflict of interest.

## STAR★Methods

### Key resources table

**Table:** 

REAGENT or RESOURCE	SOURCE	IDENTIFIER
**Antibodies**		

Anti-human CD3 (APC)	BD Bioscience	Cat# 555342; RRID: AB_398592
Anti-human CD3 (PerCP-Cy5.5)	BD Bioscience	Cat# 560835; RRID: AB_2033956
Anti-human CD4 (BV421)	BD Bioscience	Cat# 566907; RRID: AB_2739448
Anti-human CD4 (PE)	BD Bioscience	Cat# 561843; RRID: AB_395752
Anti-human CD4 (PerCP-Cy5.5)	BD Bioscience	Cat# 566316; RRID: AB_2739678
Anti-human CD5 (PE)	BD Bioscience	Cat# 555353; RRID:AB_395757
Anti-human CD7 (PE)	BD Bioscience	Cat# 555361; RRID: AB_395764
Anti-human CD7 (APC)	BD Bioscience	Cat# 561604; RRID: AB_10893354
Anti-human CD8α (FITC)	BD Bioscience	Cat# 551347; RRID: AB_394159
Anti-human CD8α (BV605)	BD Bioscience	Cat# 564116; RRID: AB_2869551
Anti-human CD8β (APC)	BD Bioscience	Cat# 641058; RRID: AB_1645723
Anti-human CD14 (FITC)	BD Bioscience	Cat# 555397; RRID: AB_395798
Anti-human CD16 (PerCP-Cy5.5)	BD Bioscience	Cat# 560717; RRID: AB_1727434
Anti-human CD19 (FITC)	BD Bioscience	Cat# 555412; RRID: AB_395812
Anti-human CD34 (FITC)	BD Bioscience	Cat# 555821; RRID: AB_396150
Anti-human CD45 (PE)	BD Bioscience	Cat# 560975; RRID: AB_395875
Anti-mouse CD45 (APC)	BD Bioscience	Cat# 561087; RRID: AB_394611
Anti-human CD56 (FITC)	BD Bioscience	Cat# 562794; RRID: AB_2737799
Anti-human CD56 (PerCP-Cy5.5)	BD Bioscience	Cat# 562794; RRID: AB_2737799
Anti-human TCRαβ (PE)	BD Bioscience	Cat# 564728; RRID: AB_2738921
Anti-human, -mouse IL-7 antibody	BioXCell	Cat# BE0048; RRID: AB_1107711

**Biological samples**		

Cord Blood from healthy donor	This paper	N/A

**Chemicals, peptides, and recombinant proteins**		

Recombinant human VEGF165	R&D Systems	Cat# 293-VE
Recombinant human BMP4	R&D Systems	Cat# 314-BP
Recombinant human SCF	R&D Systems	Cat# 300-07
Recombinant human Flt3L	R&D Systems	Cat# 300-19
Recombinant human IL-7	R&D Systems	Cat# 200-07
Recombinant human IL-15	R&D Systems	Cat# 200-15
mTeSR1	StemCell Technologies	Cat# 85850
MEMα, with nucleosides	ThermoFisher Scientific	Cat# 12571063
Fetal bovine serum, qualified, heat-inactivated, Australia	ThermoFisher Scientific	Cat# 10100147
GlutaMAX™ Supplement	ThermoFisher Scientific	Cat# 35050061
MEM Non-Essential Amino Acids Solution (100×)	ThermoFisher Scientific	Cat# 11140050
2-Mercaptoethanol, 55mM	ThermoFisher Scientific	Cat# 21985023
DMEM/F12	ThermoFisher Scientific	Cat# 11320074
TrypLE Express Enzyme (1×)	ThermoFisher Scientific	Cat# 12605010
Matrigel hESC-Qualified Matrix, LDEV-free	Corning	Cat# 354277
D-PBS, -Ca^+2^, -Mg^+2^	Sigma-Aldrich	Cat# D8537
Bovine serum albumin (BSA)	Sigma-Aldrich	Cat# B2064
Stemline® II Hematopoietic Stem Cell Expansion Medium	Sigma-Aldrich	Cat# S0192
MethoCult™ SF H4436	StemCell Technologies	Cat# 04436
Thiazovivin	GIBH CAS	N/A
Collagen IV, mouse	Corning	Cat# 354233
Penicillin-Streptomycin	Sigma-Aldrich	Cat# P4333
Hanks’ Balanced Salt Solution, w/o Ca^+2^, Mg^+2^	ThermoFisher Scientific	Cat# 14175103
HEPES (1M)	ThermoFisher Scientific	Cat# 15630080
SB-431542	Tocris Bioscience	Cat# 1614
EDTA, 0.5 M, pH 8.0	Sigma-Aldrich	Cat# 324506-100ML
Normal human serum	Sigma-Aldrich	Cat# H4522
Ficoll®-Paque PREMIUM 1.073	Sigma-Aldrich	Cat# GE17-5446-52
L-Ascorbic acid 2-phosphate sesquimagnesium salt hydrate	Sigma-Aldrich	Cat# A8960
HCl, BioReagent	Sigma-Aldrich	Cat# H1758
DNase/RNase-free deionized water	TIANGEN	Cat# RT121

**Critical commercial assays**		

CD34 MicroBead Kit, human	Miltenyi Biotec	Cat# 130-046-702

**Experimental models: Cell lines**		

OP9-DLL4	GIBH-CAS	N/A
H1 hESC	WiCell	Cat# WA01
H9 hESC	WiCell	Cat# WA09
IPS12 hiPSC	Lagarkova et al.^50^	N/A

**Experimental models: Organisms/strains**		

Mouse: NOD-scid-IL2Rg^−/−^ (NSI)	GIBH CAS	N/A

**Deposited data**		

Single cell RNA sequencing data	This paper	NCBI GEO: GSE202253

**Recombinant DNA**		

pPiggyBac-EF1α-hDLL4-Puro^R^	Philonenko et al.^15^	N/A

**Software and algorithms**		

FlowJo (V.10)	TreeStar	https://www.flowjo.com
BD FACSDiva™ Software	BD Biosciences	http://www.bdbiosciences.com
Seurat (v4.0.4)	R	https://satijalab.org/seurat/index.html
monocle3 (v1.0.0)	R	https://cole-trapnell-lab.github.io/monocle3/
Conos (v1.4.3)	R	https://github.com/kharchenkolab/conos
SeuratWrappers (v0.3.0)	R	https://github.com/satijalab/seurat-wrappers
SingleCellExperiment (v1.14.1)	R	https://github.com/drisso/SingleCellExperiment
R (v4.1.3)	R	https://www.r-project.org/
Cellranger (v4.0.0)	10× Genomics	https://www.10xgenomics.com/

### Experimental model and study participant details

#### Cell lines

hESC lines, H1/WA01, H9/WA09, and a hiPSC line, hiPSC12^50^ were maintained in the undifferentiated state on Matrigel-coated plates (Corning Matrigel, Cat. No. 354230) in mTeSR1 medium (STEMCELL Technologies, Vancouver, Canada). A single-cell suspension of hPSCs was obtained by dissociation of 70–80% confluent hPSC cultures with TrypLE Express (ThermoFisher Scientific, Waltham, MA) for a minimum time period at 37°C. Before transfection and hematopoietic differentiation, hPSCs were subjected to at least three short passages (2–3 days) at a seeding density of 4–6 × 10^6^ cells per one well of a standard 6-well plate.

#### Mice

NSI (NOD-scid-IL2Rg^−/−^) immunodeficient mice were housed and bred in the SPF-grade animal facility of the Guangzhou Institutes of Biomedicine and Health, Chinese Academy of Sciences (GIBH CAS, China). All animal experiments were approved by the Institutional Animal Care and Use Committee of Guangzhou Institutes of Biomedicine and Health (IACUC-GIBH). In these experiments, we used non-sexed newborn mice for cell injections within 24 h after the birth. The age of mice at the time of analysis is mentioned in the main text.

#### Umbilical cord blood samples

Human umbilical cord blood (CB) samples were obtained following the provision of informed consent by the child’s mother. CB mononuclear cells were obtained by density gradient centrifugation using Ficoll-Paque PREMIUM 1.073 (Sigma-Aldrich, Burlington, MA, USA). CD34^+^ CB HSPC fractions were purified by magnetic-activated cell sorting (MACS) using CD34 MicroBead Kit, human, (Miltenyi Biotec, Bergisch Gladbach, North Rhine-Westphalia, Germany) according to the manufacturer’s instructions.

### Method details

#### Hematopoietic differentiation of hPSCs

To differentiate hPSCs into hematopoietic cells, we followed a previously reported protocol^15^ with slight modifications. To induce hPSC differentiation, 1 × 10^6^ single cells were spun at 100 × g for 4 min into AggreWell400 (STEMCELL Technologies) in a mTESR1 medium containing 1 μM Thiazovivin (GIBH CAS, China) and incubated for 24 h at 37°C and 5% CO_2_. The newly formed clumps were placed on mCollagen IV (Corning Life Sciences, Bedford, MA)-coated surfaces in mTeSR1 medium supplemented with 4 ng/mL hrBMP4 (Peprotech, Rocky Hill, NJ), 50 ng/mL hrVEGF_165_ (Peprotech) and 10 μM Thiazovivin. After 48 h, the medium was replaced with StemLine II medium (Sigma-Aldrich) supplemented with GlutaMAX-I (Gibco, ThermoFisher Scientific, Waltham, MA), 1 × NEAA (Gibco), 50 μM 2-Mercaptoethanol (Sigma-Aldrich), 50 ng/mL hVEGF_165_ (Peprotech) and 6 μM SB-431542 (Tocris Bioscience, Bristol, UK). Forty-eight hours later, SB-431542 was removed from the medium. Onward, half of the medium, 2 mL in each well, was replaced with the fresh medium every second day until cell sorting. On Day 12, CD34^+^ cells were purified by MACS as described above and subjected to T cell differentiation.

#### T cell differentiation

OP9 cells transfected with a PiggyBac vector to express Delta Like Canonical Notch Ligand 4 (DLL4) were generated^15^ and maintained in the OP9 Medium containing α-MEM (Gibco) supplemented with 20% FCS (Gibco), GlutaMax-I (Gibco), 1 × NEAA (Gibco), and 50 μM 2-Mercaptoethanol (Sigma-Aldrich). CD34^+^ hematopoietic cells derived from hPSCs or UCB were seeded on pre-plated OP9-DLL4 cells in the OP9 Medium supplemented with 50 μg/mL L-Ascorbic acid 2-phosphate sesquimagnesium salt hydrate (Sigma-Aldrich), 5 ng/mL hrSCF (Peprotech), 10 ng/mL hrFlt3L (Peprotech), 10 ng/mL hrIL-7 (Peprotech). Half the media was changed every 2–3 days. After 10 days of the co-culture, non-adherent cells were re-suspended by vigorous pipetting and transferred to the new OP9-DLL4 layer, and subsequently, the transfers were performed once a week.

#### NK cell differentiation

MACS-purified Day 10 hPSC-HEC-derived CD34^+^ cells were seeded on pre-plated OP9-DLL4 cell layers in the OP9 Medium supplemented with 50 μg/mL L-Ascorbic acid 2-phosphate sesquimagnesium salt hydrate (Sigma-Aldrich), 5 ng/mL hrSCF (Peprotech), 10 ng/mL hrFlt3L (Peprotech), 10 ng/mL hrIL-7 (Peprotech), and 20 ng/mL hrIL-15 (Peprotech). Half the media was changed every 2–3 days until the day of flow cytometry analysis.

#### TCR repertoire sequencing

Total suspension cells from Day 49 T cell differentiation were collected by centrifugation and cell pellets were lyzed and frozen in TRIzol Reagent (ThermoFisher Scientific). The sequencing and analysis of TCRβ CDR3 regions were performed by Novogene (Beijing, China).

#### Flow cytometry and cell sorting

For the cell cytometry and sorting, we used anti-human monoclonal antibodies from Becton Dickinson (BD Life Sciences, Franklin Lakes, NJ). All cell sorting procedures were performed on BD FACSAria II. The flow cytometry analyses were done on BD LSRFortessa, and the data were analyzed with FlowJo V10 (FlowJo LLC, BD).

For flow cytometry analysis of emerging T cells, non-adherent and loosely adherent cells were pooled, resuspended, and cell clumps were removed using BD Falcon 40 μm Cell Strainers. The cells were centrifuged at 300 × g for 5 min at room temperature and the pellet was resuspended in the OP9 Medium. The resulting cell suspension was incubated in a CO_2_ incubator for at least 1 h to restore TrypLE-sensitive antigens. Next, the cells were centrifuged as described above and resuspended in the cold FACS Buffer (1 × D-PBS w/o Ca & Mg, 5% FCS, 20 mM HEPES pH7.2–7.5) containing 5% normal human serum at a density of 1 × 10^6^ cells per 100 μL and incubated on ice for at least 10 min. After the serum blocking, the cells were incubated with specific antibodies for 20 min on ice in the dark. The unbound antibodies were washed twice with 1 mL of the cold FACS Buffer and spun down at 300 × g for 5 min at 4°C. To exclude dead or apoptotic cells the samples were subjected to a standard DAPI staining procedure.

For sorting, cells were harvested, incubated with antibodies, and washed as described above. After the final wash, the cells were resuspended in 0.5–1 mL of the FACS Sorting Buffer (1 × HBSS (Hanks’ Balanced Salt Solution) w/o Ca & Mg, 2% BSA (Sigma-Aldrich), 25 mM HEPES pH7.2–7.5, 1 mM EDTA). The cells were filtered through a 40 μm cell strainer, sorted into the OP9-DLL4 medium, and washed twice by α-MEM before downstream applications.

#### Mouse transplantations

After 10 days of hPSC-HEC or CD34^+^ CB cell culture on OP9-DLL4 stroma, CD34^+^ cells were purified by MACS using CD34 MicroBead Kit, human, (Miltenyi Biotec). CD34^+^ cells were injected intrahepatically into Day 1–3 NSI neonates. Each mouse received 1 × 10^6^ CD34^+^ cells mixed with rhIL-7 (0.5 μg/mouse) and anti-human IL-7 mAbs, clone M25 (BioXCell, Lebanon, NH; 2.5 μg/mouse), in 30 μL in 1×D-PBS w/o Ca & Mg. Mouse engraftment was boosted with intraperitoneal injections of the IL-7/M25-mAbs cocktail every 3–4 days until the day of analysis.

#### Hematopoietic progenitor assay

Hematopoietic progenitor assay was performed in the serum-free methylcellulose medium SF H4436 (STEMCELL Technologies) according to the manufacturer’s recommendations in duplicates for at least two different cell densities of each input cell population ranging from 5 × 10^3^ to 5 × 10^4^ cells per 1.5 mL of the medium per one 35 mm Petri dish. The colonies were grown for 16–18 days at 37°C in a humidified atmosphere containing 5% CO_2_.

### Quantification and statistical analysis

#### Single cell RNA sequencing and data analysis

CD7-positive cells from Day 10 and Day 14 lymphoid cultures were sorted by FACS. Sorted cell samples were sent to Novogene (Beijing, China) for single-cell RNA sequencing. Droplet-based scRNA-seq datasets were produced using a Chromium system (10x Genomics, Pleasanton, CA). Raw reads were aligned and quantified using the CellRanger software package (version 4.0.0) and GRCh38 version of the human genome. Both sequencing experiments were processed simultaneously to merge all cells into a single count matrix. After the initial data processing, all subsequent steps were conducted using a Seurat package (version 4.0.4).^51^ Cells with a high percentage of reads from the mitochondrial genome were dropped (above 18%, the median percentage was 6.5, IQR: 5.7–7.4). Cells with a low or very high number of detected transcripts (<1000 and >7000) were also dropped (median 3133, IQR: 2540–3176). Relaxed thresholds were selected to increase sensitivity and additional quality checkups were done on the populations of interest.

Cell cycle-associated transcriptome has a pronounced influence on unsupervised clusterization and dimensional representation of cells. Therefore, after performing the data normalization with NormalizeData and scoring cell cycle genes with CellCycleScoring, we regressed the expression of cell cycle-associated genes using a built-in Seurat function SCtransform with the option vars.to.regress. For cell clustering, a kNN graph was constructed with the FindNeigbours function based on the first 20 principal components (PC). The number of PCs was defined manually based on ElbowPlot PC ranking. Cells were clustered by the FindClusters function with the resolution parameter set to 0.5 to gain biologically interpretable clusters. Finally, dimensional reduction of the resulting dataset for visualization purposes was done using the runUMAP function based on the first 20 PCs. Using the FindAllMarkers function resulting clusters were analyzed for the presence of specific markers to define the cluster identity. Specific interest was given to transcription factors^52^ and cell surface markers.^53^

To perform a comparison with the existing *in vivo* datasets we used Conos software packaged for R^30^ in combination with the SeuratWrappers package. Individual raw count matrixes of each experiment from published datasets were downloaded and individually processed to obtain normalized and filtered Seurat objects as described above for our dataset. Next, all Seurat objects were integrated into a single graph by the Conos buildGraph function with default parameters. Unsupervised clustering was performed on the resulting graph by the Leiden community algorithm with a resolution equal to 1. The dimensional reduction was performed with the UMAP procedure for visualization purposes. Larger clusters were further divided into smaller subclusters for adequate biological interpretation with the Conos function findSubcommunities.

The Conos software constructs a batch-corrected graph of shared nearest neighbors (SNN), where the vertices represent cells and the edges connect similar cells. Then, Uniform Manifold Approximation and Projection (UMAP) and Leiden approaches on this graph are employed to create joint embedding and joint clustering, respectively. They are inherently batch-corrected being based on batch-corrected graphs. The Conos graph includes edges both within samples (essentially a nearest neighbor graph) and between cells from different samples. To establish edges between cells from different samples, they are embedded in a common space.

Trajectory analysis was performed using the monocle software (version 3)^54^; the trajectory was rooted at the CD34 expressing cluster to follow the development of these cells. Surat objects generated in the previous steps were transformed by the SeuratWrapper function as.cell_data_set to monocle-compatible format, and standard trajectory analysis was done according to the monocle vignette. Circular trajectories were forbidden with the parameter close_loop set to “false” and additional trajectory smoothing was achieved by setting ncenter to 500 and rann.k to 1.

## Supplemental information

Document S1. Figures S1–S7

Document S2. Article plus supplemental information
