# Supplementary material for: Generation of thymus-reconstituting T cell progenitors from human pluripotent stem cells
Source: Cell Rep Methods. 2026 Jan 8;6(1):101272. doi: 10.1016/j.crmeth.2025.101272 (PMC12853186; doi:10.1016/j.crmeth.2025.101272)
Supplement: Document S1. Figures S1–S7 [file mmc1.pdf]

Cell Reports Methods, Volume *6*

Supplemental information

Generation of thymus-reconstituting T cell progenitors from human pluripotent stem cells

Elena S. Philonenko, Baoyun Zhang, Eugene Albert, Zahir Shah, Denis Maksimov, Yahai Shu, Peng Li, Pavel Volchkov, and Igor M. Samokhvalov

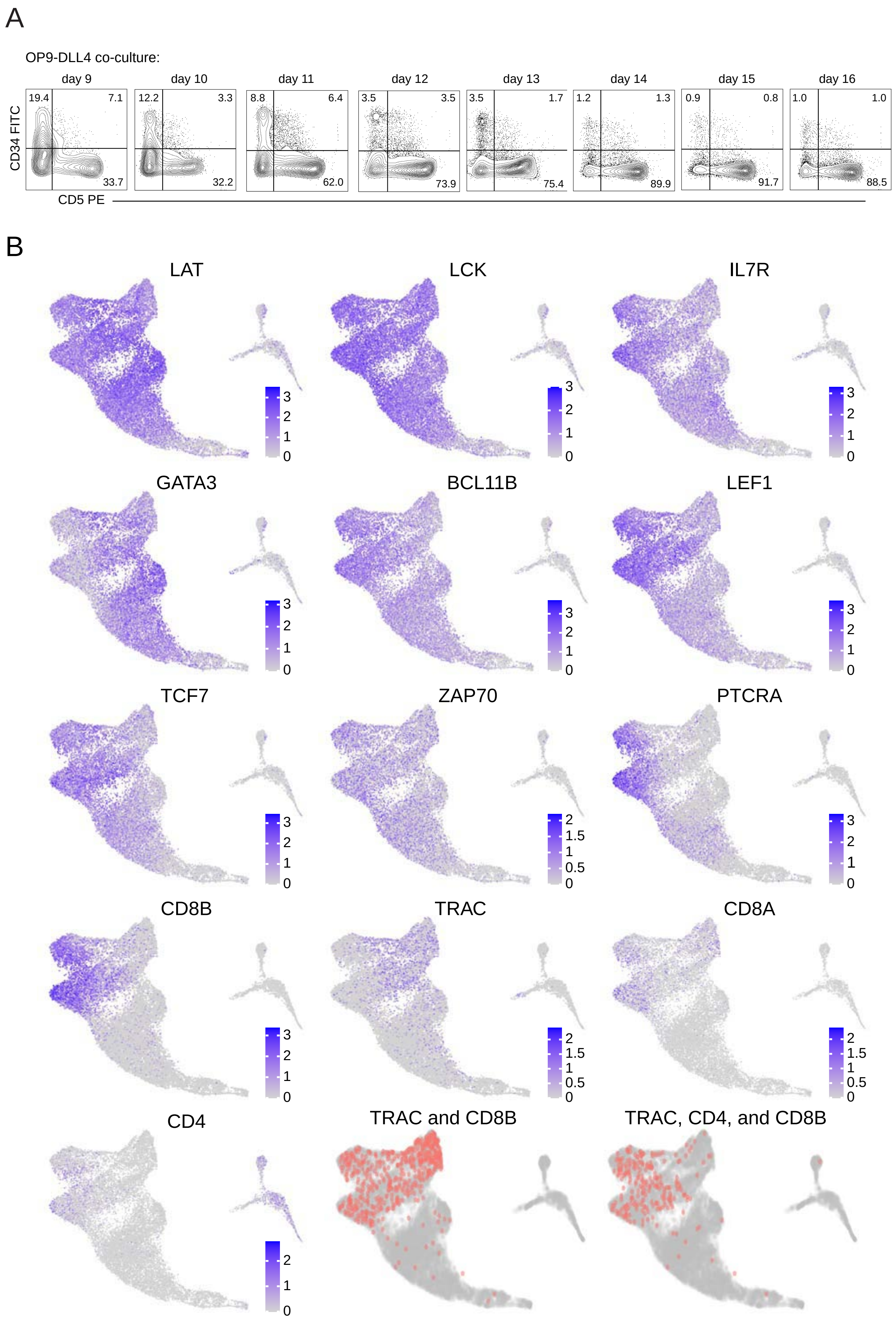

Figure S1. Rapid downregulation of CD34 during the Notch-dependent differentiation of hPSC-HECs and expression of feature lymphoid genes in Day10/Day14 CD7$^+$ cells. Related to Figures 1 and 2.

A. Flow cytometry shows a drop of CD34 expression starting from more than 26% down to 2% of live human cells between Day 9 and Day16 of the OP9-DLL4 co-culture.

B. Expression of the indicated T lineage markers projected on the UMAP vizualization. Positive cells are shown by blue dots. Scaled colors represent gene expression levels. Cells co-expressing: 1) TRAC and CD8B; 2) TRAC, CD4, and CD8; are highlighted in red.

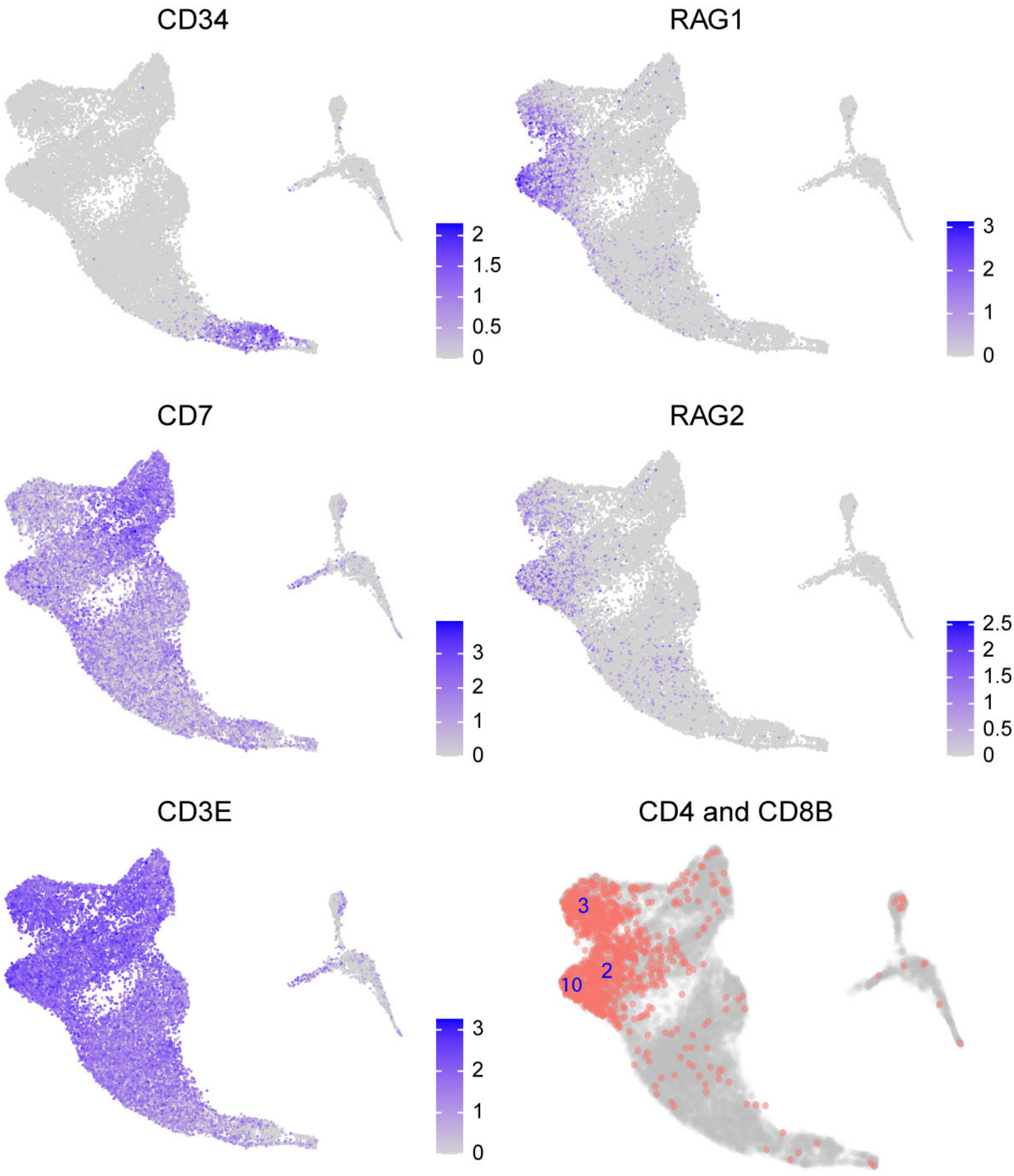

Figure S2. Expression of key lymphoid genes in Day 10/Day 14 hPSC-derived CD7^{+}cells. Related to Figure 2. Expresssion of the indicated markers projected on the UMAP visualization plot. Positive cells are shown by blue dots. Scaled colors represent the gene expression levels. Cells, co-expressing *CD4* and *CD8*, are highlighted in red. These expression patterns conceptualize three basic types of hPSC-HEC-derived T lineage cells: *CD34*$^{+}$ progenitors, *CD7*$^{+}$/*CD3ε*$^{+}$/CD4^{-}/CD8β^{-} RAG1^{-}/RAG2^{-} early T cells, and RAG1^{+}/RAG2^{+}/CD4^{+}CD8β^{+} T cells that were committed to the DP stage. Note that some CD7^{+} sorted cells stopped expressing the *CD7* gene. Clusters 2, 3, and 10 co-express *CD4* and *CD8*.

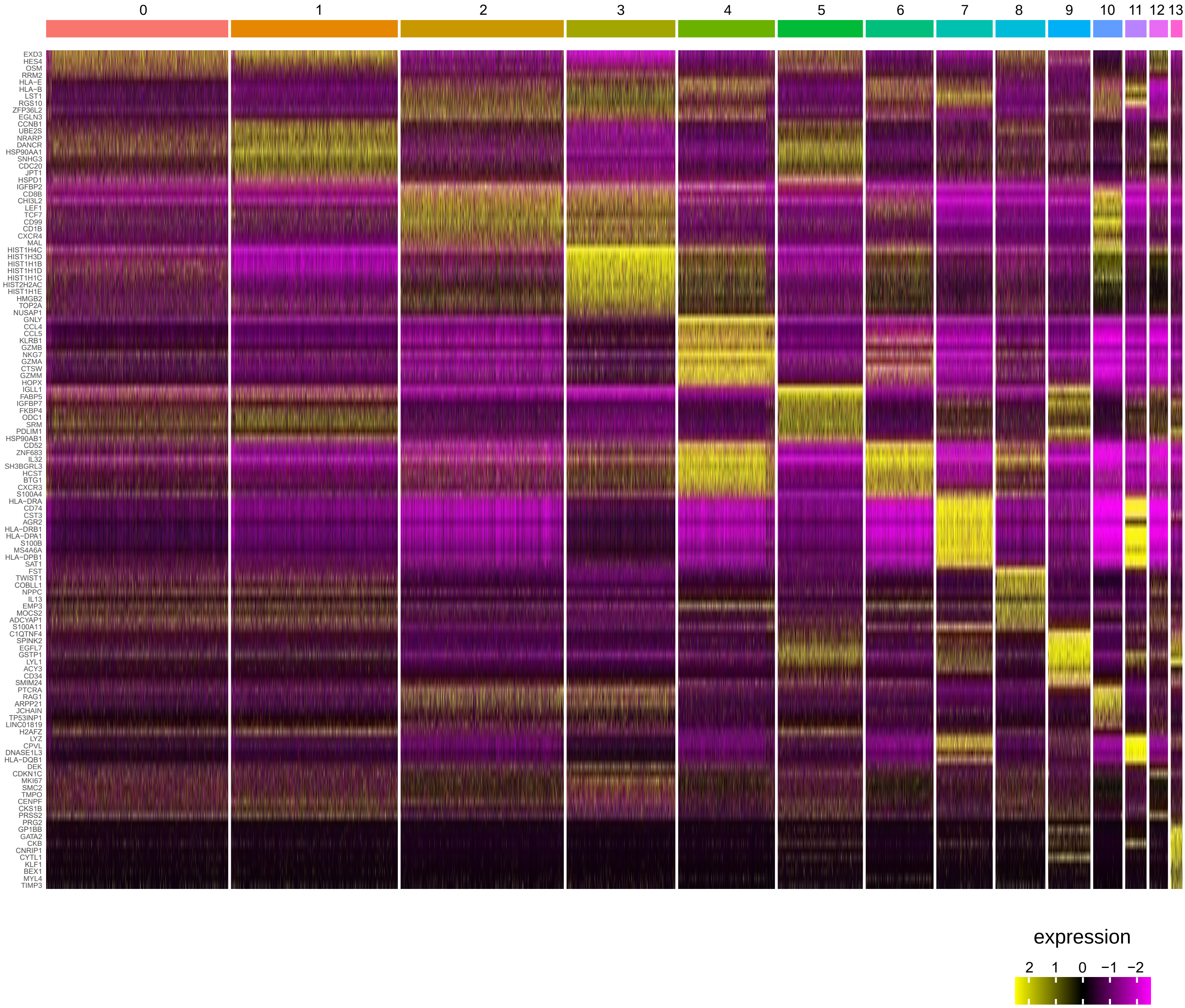

Figure S3. Heatmap of differentially expressed genes (DEGs) across the CD7^{+}cell clusters. Related to Figure 3. Each colored and numbered column represents a CD7^{+}cluster and each row represents a gene.The heatmap visualizes distinct gene expression patterns for cell clusters determined in the study.

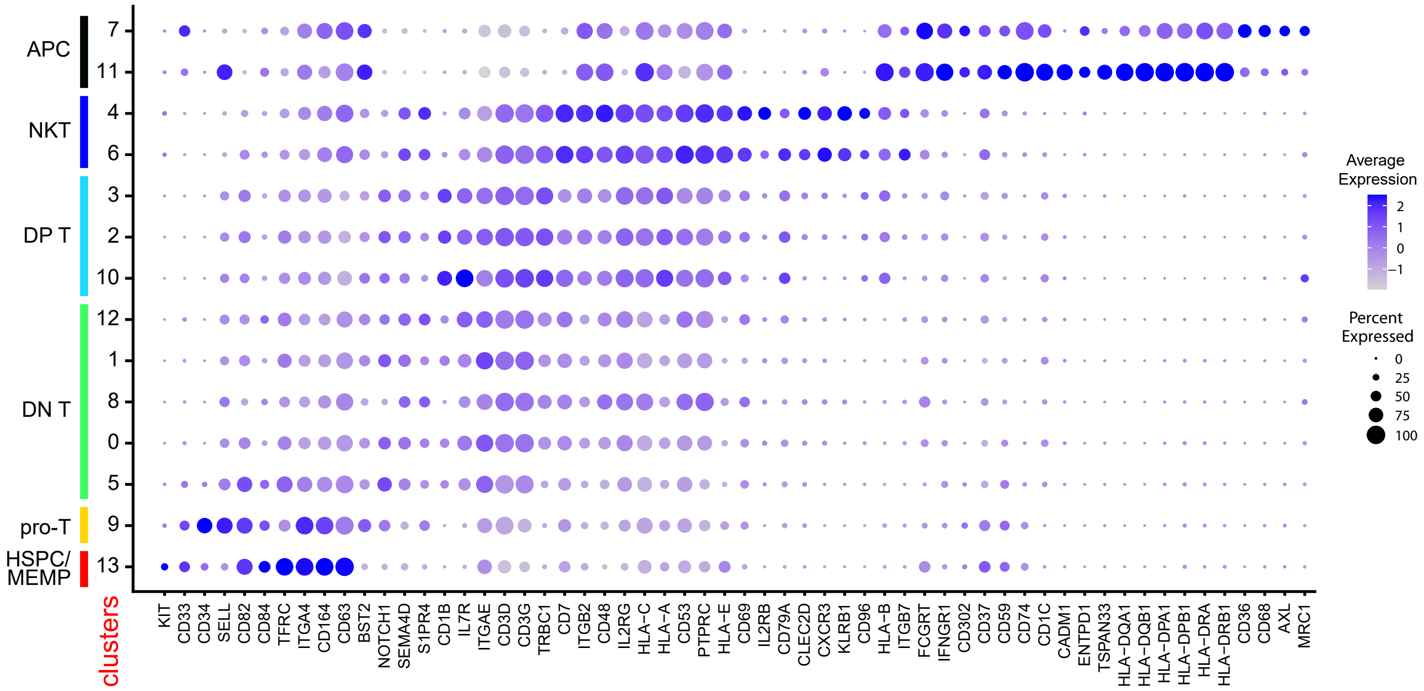

Figure S4. The expression profile of cell surface DEGs in the CD7^{+}clusters. Related to Figures 2 and 3. Scaled colors represent the gene expression levels, and dot size encodes the proportion of positive cells in the corresponding cluster. Clusters are annotated according to their transcriptome profile as shown in Figure 2E, with the same color scheme.

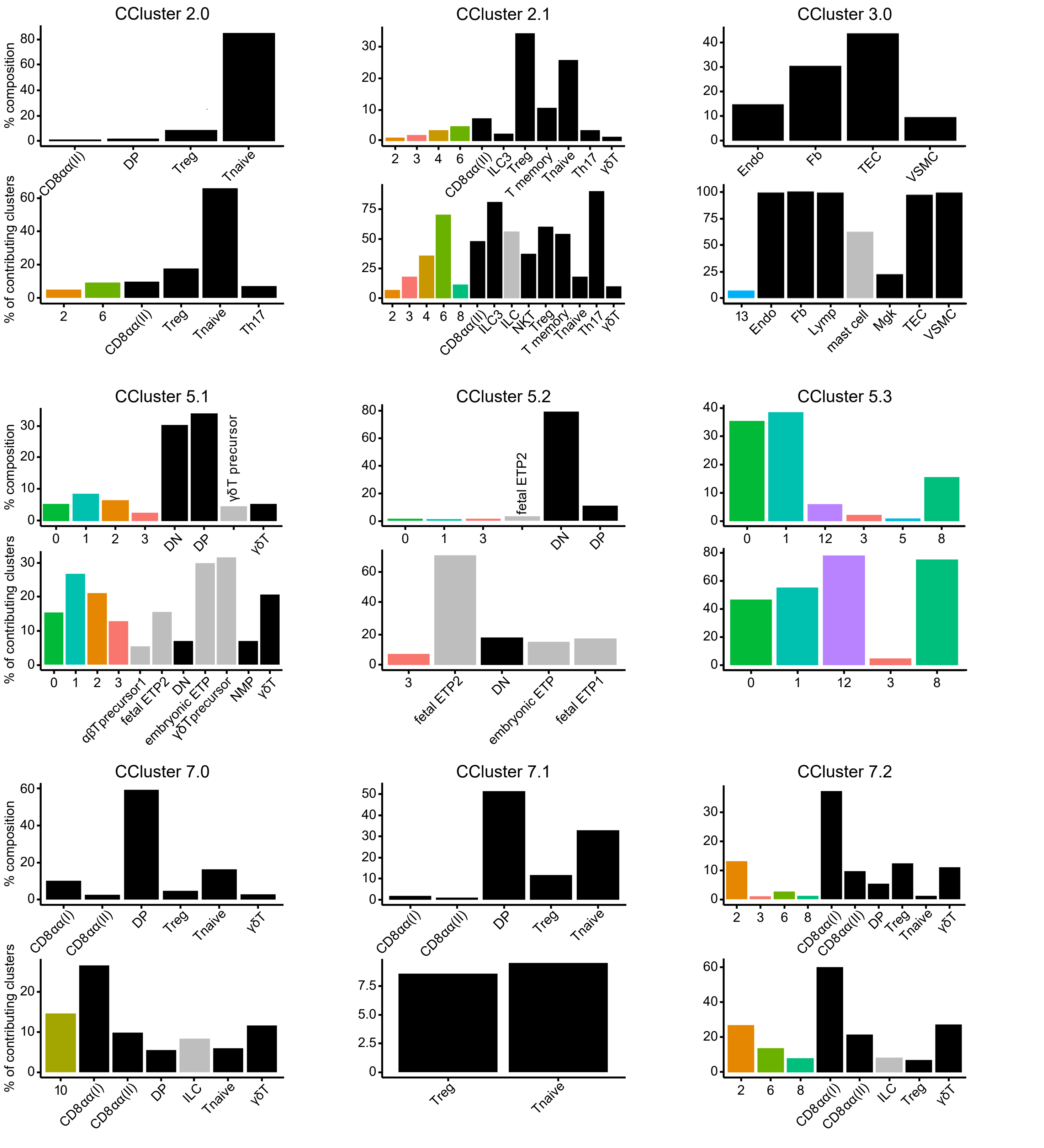

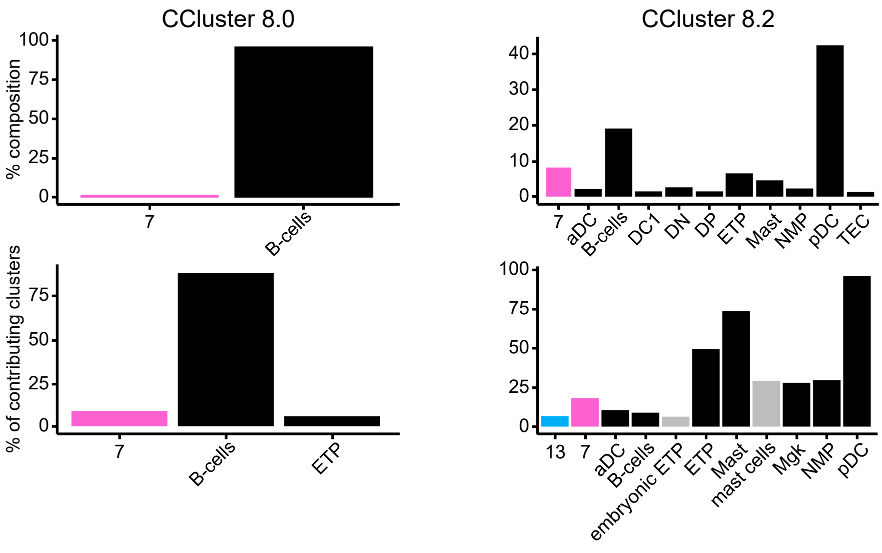

Figure S5. Characterization of an additional set of the composite cell clusters. Related to Figures 4E-L.

The location of all composite clusters is shown in Figure 4A. In the pair of bar plots for each composite cluster, the upper plot shows the percentage contribution of hPSC-HEC-derived and human thymocyte clusters to the designated composite cluster. The bottom plot shows the percentage of cells in source clusters contributing to the designated composite cluster. Colored bars represent the hPSC-HEC-derived cell clusters, black bars - Park et al., 2020 clusters, and gray bars - Zeng et al., 2019 clusters.

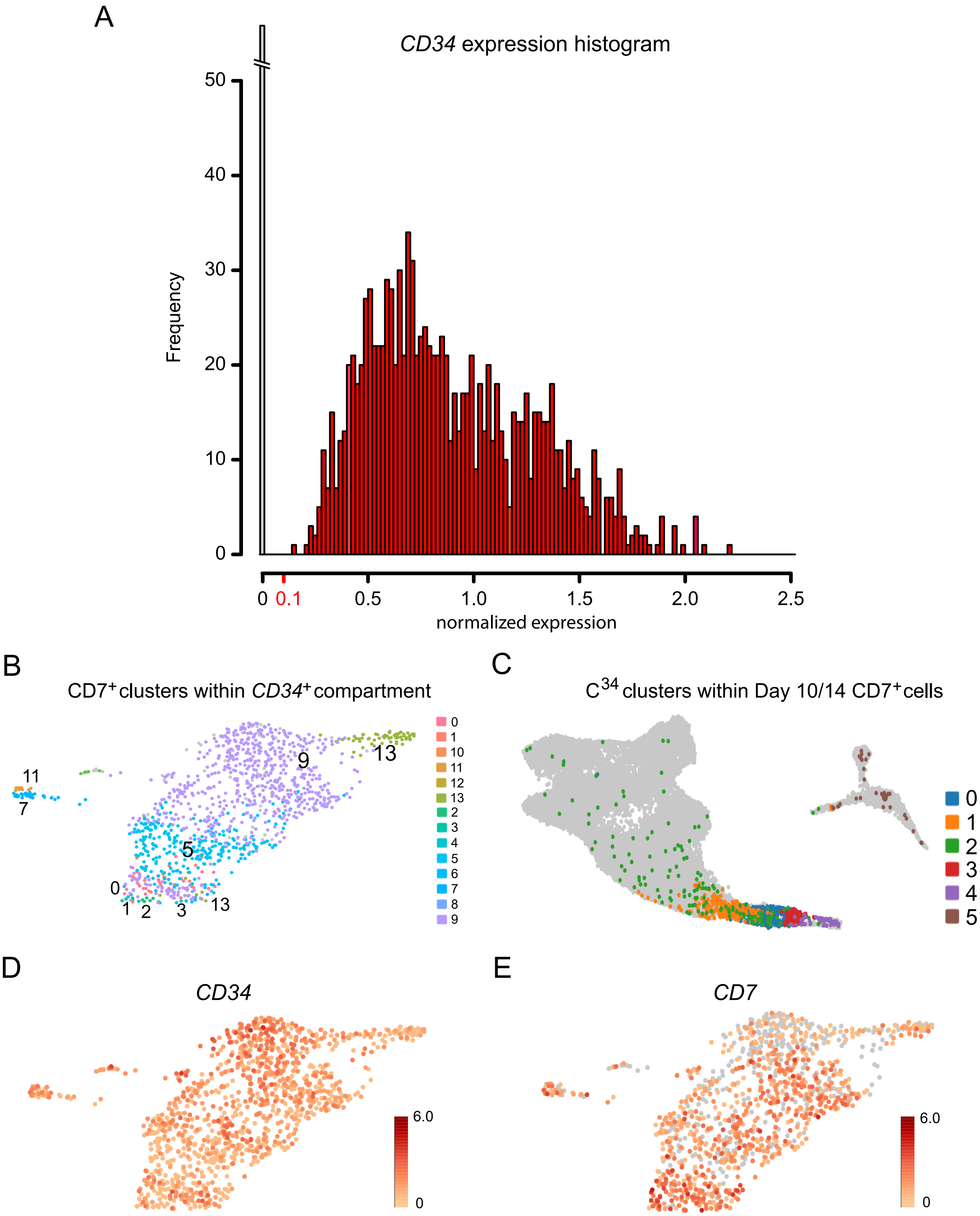

Figure S6. Characterization of the hPSC-HEC-derived *CD34*⁺ cell clusters. Related to Figure 5.
A. Cell frequency histogram of normalized *CD34* expression in the *CD34*⁺ domain.
B. UMAP visualization plot showing the contribution of CD7⁺ clusters into the *CD34*⁺ domain. Colors and numbers designate the CD7⁺ clusters.
C. Location of *CD34*⁺ clusters within the UMAP visualization of Day 10/14 CD7⁺ cells. Colors and numbers designate the *CD34*⁺ clusters.
D. Expression of *CD34* in *CD34*⁺ cells. Positive cells are shown by orange/brown dots. Scaled colors represent the gene expression levels.
E. Expression of *CD7* in *CD34*⁺ cells. Positive cells are shown by orange/brown dots. Scaled colors represent the gene expression levels.

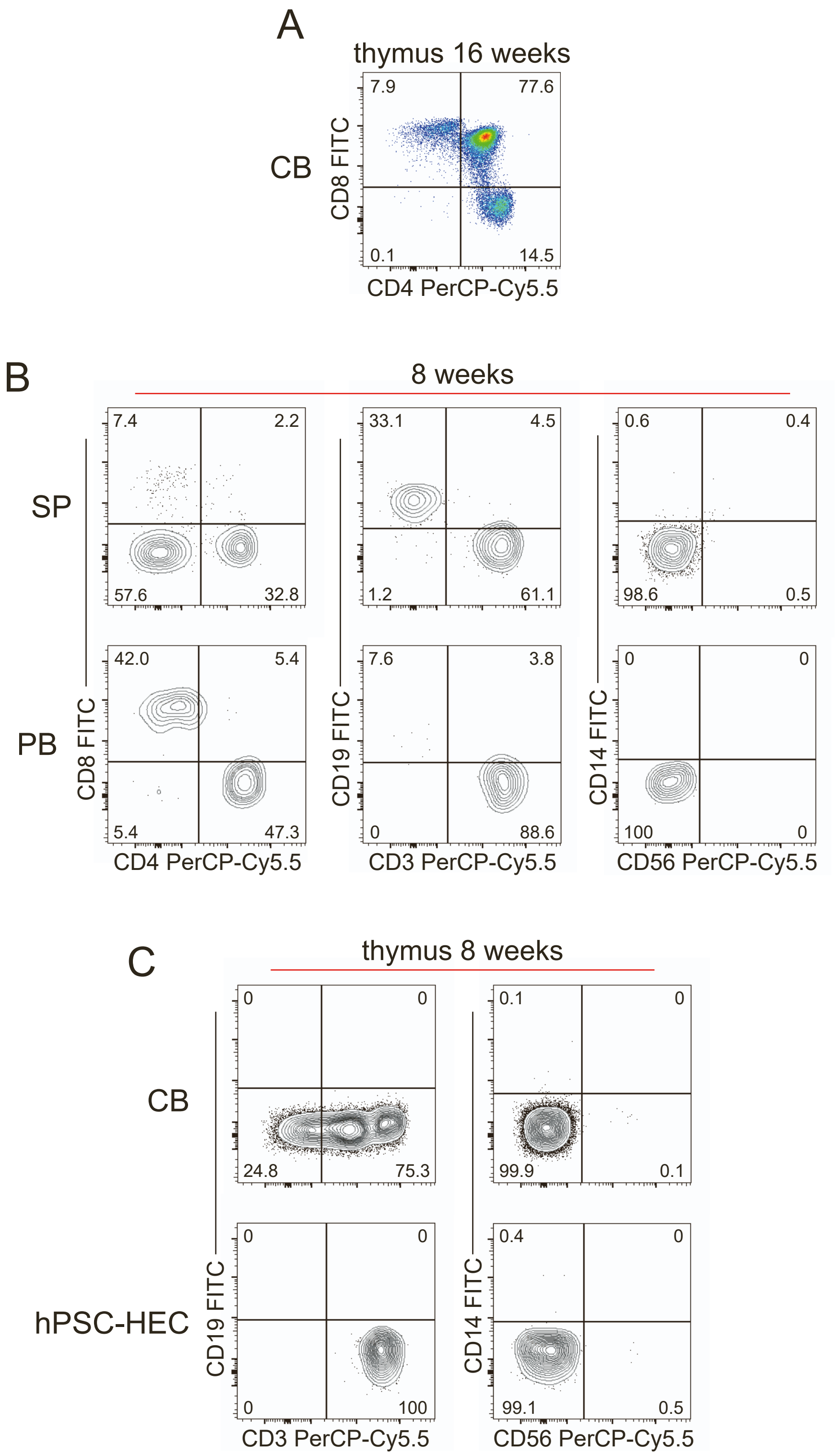

Figure S7. Mouse repopulation with cultured CD34$^+$CB cells. Related to Figure 6.

A. Flow cytometry analysis of thymocytes at 16 weeks post-transplant showing the engraftment of human DP and SP T cells.

B. Eight weeks after transplantation, cultured CD34$^+$ CB cells developed into T cells in peripheral blood and the spleen, and into B cells in the spleen, but failed to give rise to B cells in blood. No CD34$^+$CB-derived myeloid and NK cells were detected in the spleen and blood 8 weeks post-transplant.

C. Thymus engraftment by the cultured CD34$^+$ CB and CD34$^+$ hPSC-HEC-derived cells. No human myeloid, B and NK cells were detected in the reconstituted thymuses.
